# Assessing Gait & Balance in Adults with Mild Balance Impairment: G&B App Reliability and Validity

**DOI:** 10.3390/s23249718

**Published:** 2023-12-08

**Authors:** Hina Shafi, Waqar Ahmed Awan, Sharon Olsen, Furqan Ahmed Siddiqi, Naureen Tassadaq, Usman Rashid, Imran Khan Niazi

**Affiliations:** 1 Riphah College of Rehabilitation & Allied Health Sciences, Riphah International University, Islamabad 46000, Pakistan; 2 Foundation Institute of Rehabilitation Sciences, Foundation University, Islamabad 44000, Pakistan; 3 Health & Rehabilitation Research Institute, Faculty of Health & Environmental Sciences, AUT University, Auckland 1010, New Zealand; 4 Centre for Chiropractic Research, New Zealand College of Chiropractic, Auckland 1060, New Zealand; 5 Centre for Sensory-Motor Interaction (SMI), Department of Health Science and Technology, Aalborg University, 9220 Aalborg, Denmark

**Keywords:** accelerometer, balance, older adults, smartphone, reliability, validity

## Abstract

Smartphone applications (apps) that utilize embedded inertial sensors have the potential to provide valid and reliable estimations of different balance and gait parameters in older adults with mild balance impairment. This study aimed to assess the reliability, validity, and sensitivity of the Gait&Balance smartphone application (G&B App) for measuring gait and balance in a sample of middle- to older-aged adults with mild balance impairment in Pakistan. Community-dwelling adults over 50 years of age (N = 83, 50 female, range 50–75 years) with a Berg Balance Scale (BBS) score between 46/56 and 54/56 were included in the study. Data collection involved securing a smartphone to the participant’s lumbosacral spine. Participants performed six standardized balance tasks, including four quiet stance tasks and two gait tasks (walking looking straight ahead and walking with head turns). The G&B App collected accelerometry data during these tasks, and the tasks were repeated twice to assess test-retest reliability. The tasks in quiet stance were also recorded with a force plate, a gold-standard technology for measuring postural sway. Additionally, participants completed three clinical measures, the BBS, the Functional Reach Test (FRT), and the Timed Up and Go Test (TUG). Test-retest reliability within the same session was determined using intraclass correlation coefficients (ICCs) and the standard error of measurement (SEM). Validity was evaluated by correlating the G&B App outcomes against both the force plate data and the clinical measures using Pearson’s product-moment correlation coefficients. To assess the G&B App’s sensitivity to differences in balance across tasks and repetitions, one-way repeated measures analyses of variance (ANOVAs) were conducted. During quiet stance, the app demonstrated moderate reliability for steadiness on firm (ICC = 0.72) and compliant surfaces (ICC = 0.75) with eyes closed. For gait tasks, the G&B App indicated moderate to excellent reliability when walking looking straight ahead for gait symmetry (ICC = 0.65), walking speed (ICC = 0.93), step length (ICC = 0.94), and step time (ICC = 0.84). The TUG correlated with app measures under both gait conditions for walking speed (r −0.70 and 0.67), step length (r −0.56 and −0.58), and step time (r 0.58 and 0.50). The BBS correlated with app measures of walking speed under both gait conditions (r 0.55 and 0.51) and step length when walking with head turns (r = 0.53). Force plate measures of total distance wandered showed adequate to excellent correlations with G&B App measures of steadiness. Notably, G&B App measures of walking speed, gait symmetry, step length, and step time, were sensitive to detecting differences in performance between standard walking and the more difficult task of walking with head turns. This study demonstrates the G&B App’s potential as a reliable and valid tool for assessing some gait and balance parameters in middle-to-older age adults, with promise for application in low-income countries like Pakistan. The app’s accessibility and accuracy could enhance healthcare services and support preventive measures related to fall risk.

## 1. Introduction

The human life span consists of multiple stages: infancy, childhood, adulthood, middle age, and older age. With improvements in modern medicine, the number of older age people has increased significantly [1,2]. However, this change has also increased the number of individuals living with the detrimental consequences of older age [3] due to the progressive loss of normal physiological integrity [3]. The prevalence of older adults living with physical disabilities has increased [4]. In 2010, there were an estimated 101 million older adults living with severe disability, and this is expected to triple to 277 million by 2050 [3]. Furthermore, older adults from low- and middle-income regions with higher disease burdens, including cardiovascular diseases and chronic respiratory and infectious diseases, are particularly vulnerable [5]. Pakistan, which is a developing and heavily populated Southeast Asian country, has a high ratio of older versus younger adults, but fewer resources for the healthcare sector as compared with high-income countries [6]. So, the prolonged human life span, accompanied by an increase in the prevalence of physical disabilities among older adults, presents a substantial challenge for countries like Pakistan with limited healthcare resources.

Healthy older adults without severe illnesses still experience age-related changes in their neuromuscular system [4]. Changes in proprioception, vision, the vestibular system, and sensorimotor function cause impairments in static and dynamic postural control [7,8,9,10] which can lead to falling [11]. Every year one-third of older adults experience a fall, and half of these individuals experience recurrent falls and higher morbidity [12,13]. Pakistani older adults have been found to exhibit poorer balance at a relatively early age compared to individuals in high-income countries [14]. Falls not only lead to physical injury but also have psychological consequences such as fear of falling. Both of these factors can lead to difficulty with activities of daily living (ADLs), reduced community participation, and overall declined quality of life [15]. The American Geriatric Society and the Centers for Disease Control and Prevention (CDC) recommend that adults over 65 years must be screened yearly for fall risk [3]. However, the implementation of comprehensive fall risk assessment in current clinical practice is limited due to constraints on available clinical time [16]. Various clinical tools, such as the Functional reach test (FRT) [17] and the Berg Balance Scale (BBS) [18], are readily accessible and cost-effective. However, these tools possess certain limitations, including subjectivity [19] which might reduce their accuracy, and ceiling effects [20]. Computerized dynamic posturography offers quantitative measurement of center of gravity (COG) excursion and is objective and sensitive to small changes in postural control [21], but requires sophisticated and costly equipment [22] and therefore is not accessible to most clinicians. The literature also supports the use of fixed force plates to measure postural stability and motion of the center of pressure [23,24]. Force plate measurement parameters, such as increased anterior-posterior (AP) and medial-lateral (ML) displacement, have been linked to impaired balance control and fall risk in older adults [24]. However, despite the proven validity and reliability of force plates, their use outside laboratory settings is marginal [25] due to their high cost and requirement for well-trained operators [24]. Thus, to overcome these limitations, more accessible and portable tools are needed to assess balance accurately in the clinical setting [26].

In recent years, the development of lightweight, wearable inertial sensors has provided less expensive, more practical methods for the quantification of postural sway, dynamic balance, and gait [27]. These sensors, alongside newly developed algorithms, have been integrated into clinical assessments to provide automatic, quantitative measures of balance [28,29]. Examples include iSWAY, iSTEP, and iTUG [30]. Emerging technology has used both onsite testing, with the help of portable force-measuring platforms (including devices from the Nintendo Wii Balance Board, Kistler, AMTI, BTrackS, and Biodex) [31,32,33,34,35], and remote measurement, enabling clinicians to determine their patients’ balance as part of telemedicine [24]. However, despite their lower cost, these systems are not yet widely utilized.

An emerging technology for balance assessment is the use of inertial sensors embedded within standard smartphones. One example of this technology is the Gait&Balance smartphone application (G&B App) [36]. This tool has provided valid and reliable estimations of several balance and gait factors in healthy young and older adults [36,37]. In addition, the app’s sensitivity to age-related balance changes supported its validity in the healthy older adult population [37]. Previous research on the G&B App did not test a population with balance impairment and was conducted in high-income countries [36,37]. There is an apparent lack of evidence regarding smartphone measurements of balance and gait in low- to middle-income countries. Such investigations could hold significant importance in these societies, where standard clinical care may not be readily available. In these resource-limited settings, accessible and user-friendly technological measures could assist with identifying adults with balance impairment and signaling the need for intervention before falling occurs. This study explored the psychometric properties of the G&B App in older adults with mild balance impairment in Pakistan. Specifically, this study explored the test-reliability, the construct validity of the app data compared with clinical measures (Berg Balance Scale, Functional Reach Test, Time Up and Go Test), the concurrent validity of the app data compared with gold-standard force plate data, and the sensitivity of the app data to repetition (practice) and task difficulty. The alternative hypotheses were: that the test-retest reliability for G&B App outcomes would exceed ICC > 0.6, the G&B App’s outcomes would correlate significantly with both the force plate data during quiet stance tasks and the clinical measures (lower bound of 95% confidence interval of *r* > 0.5), and G&B App outcomes would be significantly different across repeated tests and tasks of increasing difficulty, in adults with mild balance impairment.

## 2. Materials and Methods

### 2.1. Study Design

This study was a single-session cross-sectional study, where reliability was determined using repeated testing, and validity was determined by comparison with gold-standard force plates (for postural sway measurement) and with clinical measures of balance.

### 2.2. Participants

The participants (N = 83) were community-dwelling older adults aged 50 years and above, including both males (N = 33) and females (N = 50), with a Berg Balance Scale (BBS) score ranging from 46 to 54 (out of 56), indicating a mild risk of falls [38] and mild balance impairment. The participants needed to be proficient in following instructions in Urdu. The exclusion criteria were individuals experiencing pain while walking, dizziness when standing or walking, any contraindications to exercise such as severe cardiovascular conditions or fractures, any contraindications related to applying a compression belt on the pelvis (e.g., pelvic surgery, compromised skin integrity), diagnosed neurological or orthopedic conditions, and musculoskeletal injuries within the past year that had impaired balance or gait. Before participating, all individuals provided written informed consent in accordance with the Declaration of Helsinki guidelines. This study was conducted with the approval of the ethical review committees of Riphah International University, Pakistan (Riphah/RCRS/REC/Letter-0011961-[7 November 2020]) and the Foundation University Islamabad (FF/FUMC/215-45 Phy20-[8 October 2020]).

### 2.3. Gait&Balance App

The G&B App is a comprehensive smartphone app that utilizes embedded inertial sensors to analyze gait and balance. This innovative app was designed to assess gait and balance during six tasks that are part of standard clinical assessments. The first four tasks focused on quiet stance balance evaluation, aiming to measure postural sway while manipulating sensory inputs crucial for maintaining balance [39]. These tasks required individuals to maintain a standing position for 30 s while (i) standing on a firm surface with eyes open (FirmEO), (ii) standing on a firm surface with eyes closed, eliminating visual information (FirmEC), (iii) standing on a compliant surface with eyes open, altering proprioceptive feedback (CompliantEO), and (iv) standing on a compliant surface with eyes closed, eliminating visual feedback and altering proprioceptive information (CompliantEC). The G&B App included auditory cues of “ready, set, go” at the beginning and end of each task, as well as a “rest” cue. The last two tasks focused on gait assessment, requiring participants (v) to walk in a straight line looking straight ahead with the head facing forwards (HF), and (vi) to walk in a straight line while turning the head from side to side (HT). For each gait task, the individual completed four short walks of six seconds each at a comfortable speed. Auditory cues of “ready, set, go” marked the beginning of each six-second walk, while a “rest, turn around” cue signaled the end. The gait assessment during these tasks is conducted over an approximate distance of 8 m (Figure S1).

### 2.4. Procedures

With the assistance of an elasticated core stability sacroiliac belt (Allcare Ortho Core Stability Belt, Whiteley Allcare, Auckland, New Zealand), which was modified to include a phone pocket specifically designed for an iPhone 7, participants were instructed to secure the belt on the lower back region. Once the sacroiliac belt was properly worn [40], participants were asked to perform the six G&B App tasks. The G&B App was utilized to collect accelerometry data. The four quiet stance tasks were performed with the feet hip-width apart, arms at the sides of the body, and shoes off for 30 s per task while standing on a gold-standard force plate (Pasco Force plate, Perform Better Limited, Southam, UK). If a participant failed to sustain the standing position, whether due to coughing, taking a step, foot movement, opening their eyes during an eyes-closed task, or requiring physical assistance, the corresponding task was terminated. The compliant surface utilized was medium-density foam (50 cm × 28 cm × 5 cm, Diamond Foam, Lahore, Pakistan). The two gait tasks were completed in an approximately 10 m hallway with a concrete floor. This set of six tasks was performed twice, with a 30 min rest period in between. Subsequently, the sacroiliac belt, along with the iPhone, was removed, and participants proceeded to perform three additional clinical balance tests: the BBS, the Functional Reach Test (FRT), and the Time Up and Go Test (TUG). The BBS evaluated balance in 14 sitting and standing tasks that required either static postures or dynamic movements and exhibited high reliability (ICC = 0.986) [41]. The BBS was scored out of 56, with higher scores indicating superior balance and lower scores indicating an increased risk of falls and poor balance [18]. The FRT was employed to assess postural stability during a reaching task and demonstrated excellent test-retest reliability in community-dwelling older adults (ICC 0.89) [17]. The FRT was carried out by calculating the maximum distance a person was able to reach forward with an outstretched arm from a standing position. The patient was instructed to flex one shoulder to 90 degrees, and then with respect to the meter scale on the wall, the position of the head of the 3rd metacarpal was noted, and the participant was asked to lean forward as much as possible without taking steps. The TUG is commonly utilized to determine balance capabilities during standing and walking movements and demonstrates excellent test-retest reliability in older adults (ICC 0.97) [42,43]. The TUG required the use of a standard chair without back support. Following instructions, the individual rose from the chair, walked at a normal pace for 3 m, executed a turn, and then returned to sit back down in the chair. The total duration required for this activity was measured using a stopwatch. 

### 2.5. Data Processing

From the quiet stance tasks, standing on a firm surface with eyes both open and closed and standing on a compliant surface with eyes both open and closed, three outcome measures were calculated: steadiness, mediolateral (ML) steadiness, and anterior-posterior (AP) steadiness. Steadiness units were reported as the negative natural logarithm of acceleration (−ln[m/s^2^]). For the gait tasks, eight outcome measures were recorded: mean walking speed (m/s), gait symmetry (periodicity index) (%), step time variability (%), average step length (m), average step time (s), step length variability (%), step length asymmetry (%), and step time asymmetry (%) [36]. All outcomes from the G&B App were calculated at two time points. 

### 2.6. Statistical Analysis

#### 2.6.1. Test-Retest Reliability

The test-retest reliability of each G&B App outcome was evaluated individually using 2-way random effects models to estimate the intra-class correlation coefficient (ICC) for absolute agreement between single measures [44,45,46]. Within the ICC model, the standard deviation of the residuals was determined and used as the standard error of the measure (SEM). To interpret the reliability, the lower bound of the 95% confidence interval (CI) of the ICC was used to classify reliability as poor, moderate, high, or excellent according to Munro (2005) [47]. 

#### 2.6.2. Validity against Clinical Measures

The validity of the G&B App outcomes against the clinical outcomes (BBS, FRT, TUG) was evaluated using Pearson’s product-moment correlation coefficient (r). App outcomes from test 2 were used for this purpose to minimize confounding caused by potential repetition effects. The magnitude of the correlation coefficient was interpreted based on the lower bound of the 95% CI of r and classified as poor, moderate, high, or excellent [48,49]. 

#### 2.6.3. Validity against Force Plate Data 

The validity of G&B App steadiness measures against the force plate outcomes was assessed using Pearson’s product-moment correlation coefficient (r). Initially, a total of 22 outcomes were computed from the force plate data using the standard pipeline provided by the force plate software (PASCO Capstone 2.0). To reduce redundancy and identify a concise set of outcome domains, a factor analysis was performed to determine the number of factors that accounted for at least 90% of the variance in the force plate outcomes. Subsequently, a single representative outcome was selected from each predetermined domain, to aid the simplicity of interpretation. These representative outcomes were then utilized to evaluate the validity of the G&B App outcomes. Furthermore, G&B App outcomes from the mediolateral (ML) and anterior-posterior (AP) directions were exclusively compared against force plate outcomes from the corresponding directions. The magnitude of the correlation coefficient was interpreted based on the 95% CI of r, and classified as poor, adequate, or excellent. [48,49]. 

#### 2.6.4. Sensitivity to Repetition Effect and Differences across Tasks

The sensitivity of the G&B App outcomes to repetition (test 1 versus test 2) and their sensitivity to the increasing difficulty across tasks was assessed with 1-way repeated measure ANOVAs with sphericity correction. For the analysis of task difficulty, data from test 2 was used, and separate analyses were conducted for quiet stance tasks (tasks 1–4) and gait tasks (tasks 5–6). For significant ANOVA results, pairwise comparisons between each of the four quiet stance tasks were performed. 

For all statistical tests, the assessment of normality and homogeneity of variance assumptions for both the data and the residuals of the model was conducted using QQ plots and fitted-vs.-residuals plots where appropriate. The statistical significance level was set at 0.05 (see the Supplementary Materials file for detailed statistical analysis). 

## 3. Results

### 3.1. Participants

The total number of enrolled participants who completed the study was 83, with an age range of 50 to 75 years (mean 56.12 ± 6.06 years). Among them, 50 were females, and the mean BMI was 28.51 ± 6.18.

### 3.2. Reliability of Gait&Balance App Measures

In terms of the balance tasks during quiet stance, G&B App measures of steadiness showed varying levels of reliability. There was moderate reliability for steadiness on a firm surface with eyes closed (ICC 0.72 [95% CI 0.60, 0.81]) and a compliant surface with eyes closed (ICC 0.75 [95% CI 0.51, 0.86]). Similarly, ML and AP steadiness on a firm surface with eyes closed exhibited moderate reliability. However, all other measures of steadiness demonstrated poor reliability (Table 1).

When assessing the gait tasks, the reliability of the G&B App’s parameters varied. For task 5 (walking with HF), parameters like walking speed, gait symmetry, step length, step time, and step time asymmetry, demonstrated moderate to excellent reliability. For task 6 (walking with HT), the G&B App’s measures pertaining to walking speed, step length, and step time exhibited high reliability. Aside from step time asymmetry when walking with HF, the reliability of step length asymmetry, step time asymmetry, step length variability, and step time variability measurements for both gait tasks was poor (Table 1).

### 3.3. Validity of Gait&Balance App with Clinical Measures 

In the four quiet stance tasks, there were no significant correlations observed between the G&B App measures of steadiness/postural stability and all three clinical measures. This lack of correlation was indicated by the 95% confidence interval (CI) of r crossing zero for all comparisons.

Table 2 presents the validity findings for the G&B App gait parameters compared with clinical measures for parameters in which the 95% CI did not cross zero. The TUG clinical measure had adequate validity against G&B App parameters of walking speed, step time, and step length when walking with head facing forwards (task 5) and walking with head-turning (task 6) (see Table 2). The BBS clinical measure had adequate validity against G&B App parameters of walking speed during both gait tasks and step length during the walking and head-turning tasks. 

### 3.4. Validity of Gait&Balance App with Force Plate Data 

A five-factor model was chosen as it accounted for 90% of the variance in the data. To facilitate further analysis, a representative outcome was selected for each domain (discussed in the method section). The force plate outcomes selected for analysis encompassed a range of parameters, each shedding light on different facets of postural control. These parameters included ‘Total ML Sway,’ which quantified cumulative side-to-side deviations in units known as ‘steps’ (representing the amount of COP movement in 1/20th of a second). Additionally, ‘Total AP Sway’ measured the sum of forward and backward deviations in the same ‘steps’ unit. ‘Total Distance Wandered’ provided a comprehensive view of overall movement by combining all sways. Furthermore, ‘Min-Step Distance’ captured the smallest individual deviations in postural sway, while ‘Max-Step Distance’ highlighted the largest observed deviations. App measures of steadiness were correlated against force plate measures of total distance wandered, min-step distance, and max-step distance. App measures of ML steadiness and AP steadiness were correlated against force plate measures of total ML sway and total AP sway, respectively. 

Table 3 presents the validity findings for the G&B App steadiness parameters compared with force plate measures. The total distance wandered exhibited an adequate to excellent correlation with app measures of steadiness across all four quiet stance tasks. Minimal step distance displayed an adequate correlation with app measures of steadiness in task 1 (firmer) and task 2 (firmEC), while maximal step distance was associated with app measures of steadiness in tasks 1, 2, and 4 (compliantEC). Furthermore, total ML sway was correlated with app measures of ML steadiness in both tasks 2 and 4 (firmEC and compliantEC). Notably, total AP sway showed a correlation with app measures of AP steadiness exclusively in task 4 (compliantEC). For tasks 3 and 4, results were variable. 

### 3.5. Sensitivity to Repetition Effect and Differences across Tasks

For the G&B App’s quiet stance tasks, the analysis of repetition (differences between test 1 and test 2) and differences across all four quiet stance tasks is shown in Table 4. 

**Figure 1:**
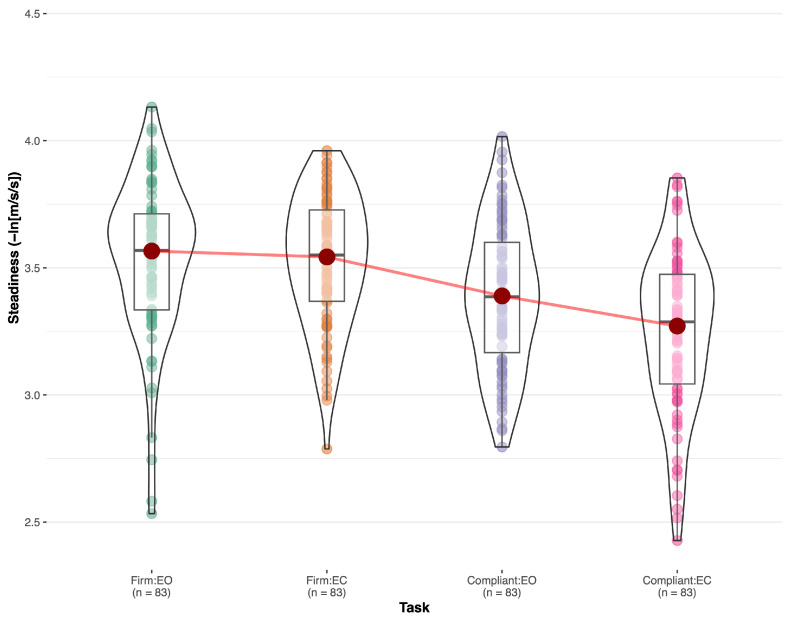
Steadiness between the four quiet stance tasks. Firm:EO = standing on a firm surface with eyes open; Firm:EC = standing on a firm surface with eyes closed (decreased visual feedback); Compliant:EO = standing on a compliant surface with eyes open (altered proprioceptive feedback); Compliant:EC = standing on a compliant surface with eyes closed (decreased visual and proprioceptive feedback).

Regarding the repetition effect in tasks 1–4, pairwise comparisons revealed significant differences between test 1 and test 2 for steadiness and ML steadiness when standing on a compliant surface with eyes open (*p* < 0.026) and eyes closed (*p* < 0.001). However, for AP steadiness, test 1 and test were not significantly different on the compliant surface with eyes open (*p* = 0.149) but were significantly different on a compliant surface with eyes closed (*p* < 0.001). For the analysis of increasing difficulty across tasks 1–4, steadiness, ML steadiness, and AP steadiness, were significantly different across the four tasks (*p* < 0.001) (Table 4). Pairwise comparisons for the steadiness outcome showed significant differences between all tasks except between task 1 (FirmEO) and task 2 (FirmEC) (see Figure 1).

For the evaluation of G&B App gait outcomes (Table 5), there were no significant effects of repetition (test 1 versus test 2). However, there were significant differences (*p* < 0.001) between parameters recorded during the less difficult walking task (walking with HF) and the more difficult walking task (walking with HT) for walking speed, gait symmetry, step length, step time, and step length variability. In contrast, no significant (*p* > 0.05) differences between HF and HT gait tasks were observed for step time asymmetry, step length asymmetry, and step time variability (Table 5). 

## 4. Discussion

This is the first study to investigate the reliability, validity, and sensitivity of the G&B App in adults with mild balance impairment. Given this smartphone app was designed for use in clinical populations with balance impairment, this research is essential to the future translation of the G&B App into clinical practice. Importantly, this study examined the use of the G&B App in a low- to middle-income country, signaling its potential for application in resource-limited settings. 

The analysis of the reliability of G&B App steadiness measures demonstrated moderate to poor reliability which is lower than previous studies. Rashid et al., (2021) [36] reported moderate to high reliability for steadiness measures in young healthy adults, and Olsen et al., 2023 [37] studied healthy middle- to older-aged adults and found moderate reliability for most steadiness measures. In the present study, participants demonstrated similar mean steadiness levels between task 1 (FirmEO) and task 2 (FirmEC), even though the second task with eyes closed was inherently more challenging than the first. Given the reduced reliability of the first task (Firm EO, ICC 0.42, 95% CI [0.23, 0.59]), this raises the possibility that participants employed a wider range of balance strategies during the first task. Since this task was less demanding, participants might not have felt the need to consistently control their stability in a particular way. In contrast, the heightened difficulty of the second task could have necessitated more consistent balancing strategies, highlighting the stricter neuromuscular control required under challenging balance conditions [50] which may have increased reliability (Firm EC, ICC 0.72, 95% CI [0.60, 0.81]). When contrasting our findings with those of Olsen et al., (2023) [37], several key differences emerge. Firstly, our study focused on a narrower age range (50–75 years), encompassing primarily older individuals who exhibited diminished balance, compared to the less impaired demographic in Olsen’s research which included some younger participants (aged 42–94 years). Thus, age-related variance in balance strategies and capability between the two studies might explain why our participants were less consistent between the two tests. In addition, the wider age band and smaller sample in Olsen et al., (2023) may have increased inter-individual variability, which raises the ICC [37]. These findings highlight the importance of exploring the reliability of G&B App steadiness measures in populations of varying ages and balance abilities. 

For the reliability of the G&B App gait measures, our study exhibited a reliability range from moderate to excellent for parameters of walking speed, step length, step time, and gait symmetry. This performance was notably slightly superior to, and more consistent than, prior studies by Rashid et al., (2021) [36] and Olsen et al., (2023) [37] for walking speed, step length, and step time parameters. This again underscores the proposition that our participants with balance impairment may have adopted more uniform balancing strategies due to the heightened neuromuscular control imperative in challenging balance scenarios [50]. Gait measures of step length variability, step time variability, step length asymmetry, and step time asymmetry had poor reliability, but this is in line with previous G&B App research [36]. 

In the validity analysis comparing G&B App measures with clinical measures, there were no correlations between of the G&B App steadiness measures (from the four quiet stance tasks) and the three clinical measures. This lack of correlation could be attributed to the nature of the clinical measures themselves, which primarily address dynamic and anticipatory balance, both within and outside the base of support (BOS) [17,41,43], and contrast with the measures of steadiness in a quiet stance position. However, when examining walking tasks, several parameters from the G&B App showed correlations with clinical measures. There was adequate validity between the G&B App parameters of walking speed, step time, step length, and the clinical TUG. This is likely because both the TUG and G&B App’s tasks 5 and 6 evaluate similar walking conditions. In addition, G&B App measures of walking speed (both while looking straight ahead and with head-turning) demonstrated correlations with the BBS. Interestingly, no such correlation was evident with the total FRT score. This differential pattern is logical as both the BBS and TUG assess dynamic gait or stepping tasks [41,43], while the FRT assesses anticipatory balance strategies with the feet fixed as the individual reaches within and beyond their BOS [17]. These findings underscore the need for further research to validate the G&B App steadiness measures against clinical measures that also assess postural sway, such as the Modified Clinical Test of Sensory Interaction in Balance (mCTSIB) [39]. Further research is especially important in clinical populations with impaired balance, where the relationship with clinical measures may be more pronounced. This may shed more light on the real-world implications and utility of these smartphone assessments.

The validity analysis of the G&B App steadiness measures compared with the gold-standard force plate parameters, such as total distance wandered, min-step distance, and max-step distance, exhibited a correlation in task 1 (FirmEO) and task 2 (FirmEC). However, the correlation between app data and force plate data for task 3 (CompliantEO) was mostly poor, which may be due to the positioning of foam over the force plate, which could alter the parameters recorded by the force plate. For task 4 (CompliantEC), the more challenging condition where greater postural sway was observed, all force plate metrics except for min-step distance correlated with app steadiness measures. Overall, these force plate findings align with a previous study that observed moderate to excellent correlations between G&B App steadiness parameters and 3D motion capture in young healthy adults [36]. The comparison of G&B App’s steadiness parameters with force plate-derived steadiness parameters revealed stronger correlations than those observed between app steadiness measures and traditional clinical measures. This discrepancy in validity findings is likely rooted in the distinct balance aspects each tool evaluates. Force plates gauge postural sway and movement of the center of pressure [24] and, therefore, naturally correlate with steadiness recorded in quiet stance with the G&B App. In contrast, clinical instruments like the BBS and TUG target more dynamic facets of gait and balance, providing a more holistic insight into multiple domains of balance [17,41,43].

In our investigation on the sensitivity of the G&B App to task difficulty, the app was able to detect the pattern of reducing steadiness levels as task difficulty increased across tasks 1 to 4. For the investigation of the effect of repetition, there was a significant difference between test 1 and test 2 for overall steadiness and ML steadiness measures during compliant surface tasks (CompliantEO, CompliantEC), suggesting a practice effect took place between tests [51]. The app’s sensitivity to these small differences in postural sway between repetitions and tasks suggests it might be responsive to small improvements in balance over time. This should be evaluated in future research. 

For the gait task evaluations, the app displayed a distinct pattern of sensitivity to differences in gait parameters under the more difficult walking task with HT. Notably, walking speed, gait symmetry, step length, step time, and step length variability all recorded significant differences between HF and HT gait tasks. This trend suggests that these parameters may be sensitive to detecting gait-related alterations during more demanding gait tasks [52]. Whereas parameters like step time variability, step length asymmetry, and step time asymmetry did not exhibit significant differences with the HT gait task. This discrepancy may originate from the inherent characteristics of the measures themselves, such as poor reliability [52,53]. 

Given the variable reliability and validity of G&B App parameters, clinicians should consider certain parameters to be more useful in clinical practice. The app is particularly adept at gauging walking speed, step length, and step time, as evidenced by their moderate to excellent reliability and correlation with trusted benchmarks like the BBS and TUG assessments. However, its scope in precisely capturing other dimensions of balance and postural stability appeared more limited in our population with mild balance impairment. For example, steadiness measures had adequate correlations with gold-standard force plate data but had poor reliability in the eyes-open tasks (FirmEO, CompliantEO). This study, being conducted in Pakistan, adds novelty, suggesting that finding valid measures in such a distinct environment enhances the app’s generalizability across various contexts. As we move forward, research should probe deeper into users’ balancing strategies across different tasks and validate the app in diverse clinical groups. The connection between the app’s steadiness parameters and force plate data remains promising, suggesting the app could provide clinicians with measures of postural sway that are not currently available in clinical practice. This dual understanding of the app’s strengths and limitations provides valuable insights for future investigations.

## 5. Conclusions

The varying reliability and validity of the G&B App across different gait and balance measures suggests its potential as a supplementary tool to clinical assessments. Notably, the app excels in specific areas, particularly in measuring walking speed, step length, and step time, as emphasized by the alignment of these parameters with established clinical benchmarks and their moderate to excellent reliability. The app could be particularly valuable for recording measures of step length and step time which are not typically available in standard practice. However, gaps remain, especially concerning the reliable assessment of steadiness, step length variability, step time variability, step length asymmetry, and step time asymmetry. The G&B App steadiness data correlated with gold-standard force plate data and was sensitive to small improvements due to practice and the decline in balance as tasks became more difficult. This suggests the app might be responsive to small changes over time, such as those that occur during rehabilitation, which should be evaluated in continued research. Our examination of the G&B App within the context of a Pakistani cohort offers both novel insights and challenges. The geographical uniqueness of our study, situated in Pakistan, underscores the importance of contextual evaluations and bolsters the app’s potential generalizability to diverse environments. As we seek to refine and enhance such digital tools, understanding their limitations and strengths within specific cultural and demographic contexts will be paramount.

## Figures and Tables

**Table 1:** Reliability of G&B App outcomes for all tasks.

Outcome	Task Qual		Test Mean ± SD		SEM	SEM%	ICC [95% CI]	Reliability
			Test-1	Test-2				
Steadiness (−ln[m/s^2^])	Firm	EO	3.56 ± 0.32	3.54 ± 0.31	0.2	7	0.43 [0.23, 0.59]	Poor
		EC	3.54 ± 0.27	3.52 ± 0.26	0.1	4	0.72 [0.6, 0.81]	Moderate
	Compliant	EO	3.33 ± 0.29	3.39 ± 0.29	0.2	5	0.62 [0.47, 0.74]	Poor
		EC	3.13 ± 0.32	3.25 ± 0.32	0.1	4	0.75 [0.51, 0.86]	Moderate
ML steadiness (−ln[m/s^2^])	Firm	EO	4.22 ± 0.37	4.21 ± 0.35	0.3	7	0.38 [0.18, 0.55]	Poor
		EC	4.26 ± 0.31	4.25 ± 0.29	0.2	4	0.71 [0.59, 0.8]	Moderate
	Compliant	EO	3.93 ± 0.31	4.04 ± 0.32	0.2	5	0.56 [0.37, 0.7]	Poor
		EC	3.8 ± 0.35	3.94 ± 0.35	0.2	4	0.7 [0.45, 0.83]	Poor
AP steadiness (−ln[m/s^2^])	Firm	EO	4.17 ± 0.28	4.14 ± 0.29	0.2	5	0.52 [0.35, 0.66]	Poor
		EC	4.08 ± 0.28	4.07 ± 0.26	0.2	4	0.69 [0.56, 0.79]	Moderate
	Compliant	EO	3.97 ± 0.3,	4.01 ± 0.29	0.2	5	0.6 [0.45, 0.72]	Poor
		EC	3.69 ± 0.32	3.81 ± 0.31	0.2	4	0.71 [0.49, 0.83]	Poor
Walking speed (m/s)	Walking	HF	0.82 ± 0.19	0.85 ± 0.2	0.05	6	0.93 [0.89, 0.96]	High
Gait symmetry (%)			64 ± 5	64 ± 5	3.00	5	0.65 [0.5, 0.76]	Moderate
Step length (m)			0.57 ± 0.07	0.57 ± 0.07	0.02	3	0.94 [0.91, 0.96]	Excellent
Step time (s)			0.64 ± 0.08	0.63 ± 0.08	0.03	5	0.84 [0.76, 0.89]	High
Step length variability (%)			6 ± 4	6 ± 6	4.00	77	0.16 [−0.06, 0.36]	Poor
Step time variability (%)			7 ± 4	6 ± 5	3.00	47	0.51 [0.33, 0.65]	Poor
Step length asymmetry (%)			4 ± 4	4 ± 4	2.00	56	0.6 [0.44, 0.72]	Poor
Step time asymmetry (%)			4 ± 4	5 ± 4	2.00	47	0.68 [0.55, 0.78]	Moderate
Walking speed (m/s)	Walking	HT	0.91 ± 0.18	0.91 ± 0.18	0.06	6	0.89 [0.84, 0.93]	High
Gait symmetry (%)			59 ± 7	60 ± 7	4.00	7	0.63 [0.47, 0.74]	Poor
Step length (m)			0.54 ± 0.08	0.55 ± 0.09	0.02	4	0.92 [0.88, 0.95]	High
Step time (S)			0.67 ± 0.09	0.66 ± 0.09	0.04	5	0.84 [0.76, 0.89]	High
Step length variability (%)			7 ± 4	7 ± 6	4.00	62	0.25 [0.03, 0.44]	Poor
Step time variability (%)			8 ± 5	7 ± 5	4.00	54	0.4 [0.21, 0.57]	Poor
Step length variability (%)			5 ± 4	5 ± 4	3.00	62	0.59 [0.44, 0.72]	Poor
Step time variability (%)			5 ± 5	5 ± 5	3.00	66	0.52 [0.35, 0.66]	Poor

SEM = standard error of measurement expressed in the outcome units; ICC = intraclass correlation coefficient; SEM% = percentage representation of the standard error of measurement in relation to the mean of the outcome; EO = eyes open; EC = eyes closed; HF = head forward; HT = head turning.

**Table 2:** Validity of G&B App outcomes against clinical measures.

Task	Outcome	Clinical Outcome	r [95% CI]	Correlation
HF	Walking speed (m/s)	BBS	0.55 [0.38, 0.69]	(+)Adequate
	Walking speed (m/s)	TUG	−0.7 [−0.79, −0.57]	(−)Adequate
	Gait symmetry (%)	BBS	0.27 [0.05, 0.46]	(+)Poor
	Gait symmetry (%)	TUG	−0.43 [−0.59, −0.24]	(−)Poor
	Step length (m)	BBS	0.45 [0.26, 0.6]	(+)Poor
	Step length (m)	FRT	0.22 [0.01, 0.42]	(+)Poor
	Step length (m)	TUG	−0.56 [−0.69, −0.39]	(−)Adequate
	Step time (s)	BBS	−0.47 [−0.62, −0.28]	(−)Poor
	Step time (s)	TUG	0.58 [0.42, 0.71]	(+)Adequate
	Step time variability (%)	BBS	−0.26 [−0.45, −0.05]	(−)Poor
	Step time variability (%)	TUG	0.31 [0.1, 0.49]	(+)Poor
	Step time asymmetry (%)	FRT	0.23 [0.02, 0.43]	(+)Poor
HT	Walking speed (m/s)	BBS	0.51 [0.33, 0.66]	(+)Adequate
	Walking speed (m/s)	TUG	−0.67 [−0.77, −0.53]	(−)Excellent
	Gait symmetry (%)	BBS	0.41 [0.21, 0.57]	(+)Poor
	Gait symmetry (%)	TUG	−0.47 [−0.62, −0.28]	(−)Poor
	Step length (m)	BBS	0.53 [0.36, 0.67]	(+)Adequate
	Step length (m)	TUG	−0.58 [−0.7, −0.41]	(−)Adequate
	Step time (s)	BBS	−0.26 [−0.45, −0.05]	(−)Poor
	Step time (s)	TUG	0.5 [0.32, 0.65]	(+)Adequate
	Step length variability (%)	BBS	−0.38 [−0.55, −0.18]	(−)Poor
	Step length variability (%)	TUG	0.33 [0.12, 0.51]	(+)Poor
	Step time variability (%)	BBS	−0.47 [−0.63, −0.29]	(−)Poor
	Step time variability (%)	TUG	0.46 [0.28, 0.62]	(+)Poor
	Step length asymmetry (%)	BBS	−0.37 [−0.54, −0.17]	(−)Poor
	Step time asymmetry (%)	BBS	−0.31 [−0.49, −0.1]	(−)Poor
	Step time asymmetry (%)	TUG	0.23 [0.01, 0.42]	(+)Poor

CI = confidence intervals. (+) = positive correlation; (−) negative Correlation.

**Table 3:** Validity of Gait&Balance App outcomes for quiet stance tasks against force plate.

Task (Number)	Qual	Outcome	FP Outcome	r [95% CI]	Correlation
Firm (tasks 1&2)	EO	Steadiness	Total distance wandered	−0.7 [−0.8, −0.55]	(−)Adequate
			Min-step distance	−0.54 [−0.68, −0.35]	(−)Adequate
			Max-step distance	−0.53 [−0.68, −0.34]	(−)Adequate
		ML steadiness	Total ML sway	−0.47 [−0.64, −0.27]	(−)Poor
		AP steadiness	Total AP sway	−0.34 [−0.53, −0.11]	(−)Poor
	EC	Steadiness	Total distance wandered	−0.83 [−0.89, −0.73]	(−)Excellent
			Min-step distance	−0.57 [−0.71, −0.39]	(−)Adequate
			Max-step distance	−0.7 [−0.8, −0.55]	(−)Adequate
		ML steadiness	Total ML sway	−0.6 [−0.73, −0.42]	(−)Adequate
		AP steadiness	Total AP sway	−0.46 [−0.63, −0.25]	(−)Poor
Compliant (tasks 3&4)	EO	Steadiness	Total distance wandered	−0.73 [−0.82, −0.59]	(−)Adequate
			Min-step distance	−0.31 [−0.51, −0.08]	(−)Poor
			Max-step distance	−0.02 [−0.26, 0.22]	(−)Poor
		ML steadiness	Total ML sway	−0.31 [−0.51, −0.08]	(−)Poor
		AP steadiness	Total AP sway	−0.15 [−0.37, 0.09]	(−)Poor
	EC	Steadiness	Total distance wandered	−0.9 [−0.94, −0.85]	(−)Excellent
			Min-step distance	−0.29 [−0.49, −0.05]	(−)Poor
			Max-step distance	−0.83 [−0.89, −0.74]	(−)Excellent
		ML steadiness	Total ML sway	−0.59 [−0.72, −0.41]	(−)Adequate
		AP steadiness	Total AP sway	−0.58 [−0.72, −0.4]	(−)Adequate

EO = eyes open; EC = eyes closed; CI = confidence intervals; FP = force plate: AP = anteroposterior; ML = mediolateral. (−) Negative Correlation; Unit for steadiness = −ln[m/s^2^].

**Table 4:** Sensitivity to repetition and differences in difficulty for quiet stance tasks.

Outcome	Task	Qual	Mean ± SD (Test 1, Test 2)	RmANOVA, *p*-Value
Steadiness	FirmEO	EO	3.56 ± 0.32, 3.54 ± 0.31	F(1, 82) = 0.35, 0.555
	FirmEC	EC	3.54 ± 0.27, 3.52 ± 0.26	F(1, 82) = 0.89, 0.347
	CompliantEO	EO	3.33 ± 0.29, 3.39 ± 0.29	F(1, 82) = 5.17, 0.026
	CompliantEC	EC	3.13 ± 0.32, 3.25 ± 0.32	F(1, 82) = 27.43, <0.001
	Between the above 4 tasks			F(2.45, 201.29) = 46.67, <0.001
ML steadiness	FirmEO	EO	4.22 ± 0.37, 4.21 ± 0.35	F(1, 82) = 0.07, 0.797
	FirmEC	EC	4.26 ± 0.31, 4.25 ± 0.29	F(1, 82) = 0.09, 0.761
	CompliantEO	EO	3.93 ± 0.31, 4.04 ± 0.32	F(1, 82) = 12.79, 0.001
	CompliantEC	EC	3.8 ± 0.35, 3.94 ± 0.35	F(1, 82) = 27.96, <0.001
	Between the above 4 tasks			F(2.38, 195.31) = 36.74, <0.001
AP steadiness	FirmEO	EO	4.17 ± 0.28, 4.14 ± 0.29	F(1, 82) = 1.02, 0.316
	FirmEC	EC	4.08 ± 0.28, 4.07 ± 0.26	F(1, 82) = 0.58, 0.448
	CompliantEO	EO	3.97 ± 0.3, 4.01 ± 0.29	F(1, 82) = 2.12, 0.149
	CompliantEC	EC	3.69 ± 0.32, 3.81 ± 0.31	F(1, 82) = 22.63, <0.001
	Between the above 4 tasks			F(2.61, 214.1) = 54.25, <0.001

Unit for steadiness = −ln[m/s^2^].

**Table 5:** Sensitivity to repetition and differences in difficulty for gait outcomes.

Outcome	Qual	Mean ± SD (Test 1, Test 2)	RmANOVA, *p*-Value
Walking speed (m/s)	HF	0.91 ± 0.18, 0.91 ± 0.18	F(1, 82) = 0.71, 0.402
	HT	0.82 ± 0.19, 0.85 ± 0.2	F(1, 82) = 10.4, 0.002
	Between the above 2 tasks		F(1, 82) = 51.78, <0.001
Gait symmetry (%)	HF	64 ± 5, 64 ± 5	F(1, 82) = 0.11, 0.739
	HT	59 ± 7, 60 ± 7	F(1, 82) = 0.97, 0.327
	Between the above 2 tasks		F(1, 82) = 48.29, <0.001
Step length (m)	HF	0.57 ± 0.07, 0.57 ± 0.07	F(1, 82) = 0.23, 0.631
	HT	0.54 ± 0.08, 0.55 ± 0.09	F(1, 82) = 1.86, 0.176
	Between the above 2 tasks		F(1, 82) = 36.88, <0.001
Step time (s)	HF	0.64 ± 0.08, 0.63 ± 0.08	F(1, 82) = 0.57, 0.453
	HT	0.67 ± 0.09, 0.66 ± 0.09	F(1, 82) = 2.41, 0.125
	Between the above 2 Tasks		F(1, 82) = 18.37, <0.001
Step length variability (%)	HF	6 ± 4, 6 ± 6	F(1, 82) = 0, 0.997
	HT	7 ± 4, 7 ± 6	F(1, 82) = 0.01, 0.93
	Between the above 2 tasks		F(1, 82) = 11.56, 0.001
Step time variability (%)	HF	7 ± 4, 6 ± 5	F(1, 82) = 0.05, 0.829
	HT	8 ± 5, 7 ± 5	F(1, 82) = 0.12, 0.729
	Between the above 2 tasks		F(1, 82) = 3.44, 0.067
Step length asymmetry (%)	HF	4 ± 4, 4 ± 4	F(1, 82) = 1.09, 0.299
	HT	5 ± 4, 5 ± 4	F(1, 82) = 1.29, 0.26
	Between the above 2 tasks		F(1, 82) = 0.08, 0.782
Step time asymmetry (%)	HF	4 ± 4, 5 ± 4	F(1, 82) = 1.79, 0.184
	HT	5 ± 5, 5 ± 5	F(1, 82) = 0.89, 0.349
	Between the above 2 tasks		F(1, 82) = 1.89, 0.173

## Data Availability

Requests for data can be made to the corresponding author, but approval from the ethics committee is required before any data can be shared.

## Supplementary Materials

The following supporting information can be downloaded at: https://www.mdpi.com/article/10.3390/s23249718/s1, Detailed statistical analysis along with App screenshot (Figure S1). Reference [54] is cited in the Supplementary Materials.
