# Supplementary material for: Assessing Gait & Balance in Adults with Mild Balance Impairment: G&B App Reliability and Validity
Source: Sensors (Basel). 2023 Dec 8;23(24):9718. doi: 10.3390/s23249718 (PMC10747653; doi:10.3390/s23249718)
Supplement: Supplementary file 1 [file sensors-23-09718-s001.zip › sensors-2711223-supplementary.pdf]

Assessing Gait & Balance in Adults with Mild Balance Impairment: G&B App Reliability and Validity

Usman Rashid

20/11/2023

Contents

1 Outcome Measures **3**
1.1 Gait Tasks . . . 3
1.2 Static Tasks . . . 3

2 Statistical Analysis **3**
2.1 Test-retest Reliability . . . 3
2.2 Validity against Clinical Outcomes . . . 3
2.3 Validity against Force Plate Data . . . 3
2.4 Responsiveness to Repetition Effect and Differences across Tasks . . . 4
2.5 Assumptions . . . 4

3 Results **5**
3.1 Reliability of Gait Outcomes . . . 5
3.2 Reliability of Outcomes for Static Tasks . . . 6
3.3 Validity against Clinical Outcomes . . . 7
3.3.1 Validity of Gait Outcomes . . . 7
3.3.2 Validity of Outcomes for Static Tasks . . . 8
3.4 Validity againt Force Plate Data . . . 9
3.4.1 Selection of Force Plate Outcomes . . . 9
3.4.2 Validity of Outcomes for Static Tasks . . . 11
3.5 Responsiveness to Repetition Effect and Task Differences . . . 12
3.5.1 Responsiveness of Gait Outcomes . . . 12
3.5.2 Responsiveness of Outcomes for Static Tasks . . . 13

4 Additional Results **15**
4.1 Model Residuals: Reliability of Gait Outcomes . . . 15
4.1.1 Gait.symmetry, HF . . . 15
4.1.2 Step.length, HF . . . 16
4.1.3 Step.time, HF . . . 16
4.1.4 Step.length.var, HF . . . 17
4.1.5 Step.time.var, HF . . . 17
4.1.6 Step.length.asym, HF . . . 18
4.1.7 Step.time.asym, HF . . . 18
4.1.8 Step.velocity, HF . . . 19
4.1.9 Gait.symmetry, HT . . . 19
4.1.10 Step.length, HT . . . 20
4.1.11 Step.time, HT . . . 20

4.1.12 Step.length.var, HT . . . 21
4.1.13 Step.time.var, HT . . . 21
4.1.14 Step.length.asym, HT . . . 22
4.1.15 Step.time.asym, HT . . . 22
4.1.16 Step.velocity, HT . . . 23
4.2 Model Residuals: Reliability of Outcomes for Static Tasks . . . 24
4.2.1 Steadiness, Firm EO . . . 24
4.2.2 Steadiness, Firm EC . . . 25
4.2.3 Steadiness, Compliant EO . . . 25
4.2.4 Steadiness, Compliant EC . . . 26
4.2.5 Steadiness.ML, Firm EO . . . 26
4.2.6 Steadiness.ML, Firm EC . . . 27
4.2.7 Steadiness.ML, Compliant EO . . . 27
4.2.8 Steadiness.ML, Compliant EC . . . 28
4.2.9 Steadiness.AP, Firm EO . . . 28
4.2.10 Steadiness.AP, Firm EC . . . 29
4.2.11 Steadiness.AP, Compliant EO . . . 29
4.2.12 Steadiness.AP, Compliant EC . . . 30

References **31**

1 Outcome Measures

1.1 Gait Tasks

There are eight outcome measures: Periodicity (Gait Symmetry Index), Average Step Length (SL_{Av}), Average Step Time (ST_{Av}), Step Length Variability (SL_{Vr}), Step Time Variability (ST_{Vr}), Step Length Asymmetry (SL_{As}), Step Time Asymmetry (ST_{As}) and Walking Speed (Average Step Velocity, WS). There are two tasks that a participant performs: Comfortable gait with head forwards (HF) and head turning (HT). Each task is performed 2 times. All the outcomes are in S.I. units.

1.2 Static Tasks

There are three outcome measures: steadiness, steadiness mediolateral (ML), steadiness anterior-posterior (AP). There are four tasks that a participant performs: standing on firm surface with eyes open/closed, and standing on compliant surface with eyes open/closed. Each task is performed 2 times. Units of steadiness are negative natural log of acceleration (-ln[m/s/s]).

2 Statistical Analysis

2.1 Test-retest Reliability

The test-retest reliability of each outcome is assessed separately using 2-way random effects models to estimate intra-class correlation coefficient (ICC) for absolute agreement between single measures (Koo & Li, 2016; Nakagawa, Johnson, & Schielzeth, 2017; Nakagawa & Schielzeth, 2010). From the ICC model, the standard deviation of the residuals is taken as the standard error of the measure (SEM). SEM is interpreted as the error associated with a single measurement taken on a random day. SEM is also expressed as percentage of test 2 mean ($SEM_{\%}$). The mean of the second test is used for $SEM\%$ calculation as it is expected to have minimal repetition effect. Reliability interpretation is based on the lower bound for the 95% confidence interval of the ICC as follows (Munro, 2005): excellent (0.90–1), high (0.7–0.89), moderate (0.50–0.69), and poor (0–0.49).

2.2 Validity against Clinical Outcomes

Validity against the clinical outcomes (BBS, FRT, TUG) is evaluated using Pearson's product-moment correlation coefficient (r). App outcomes from test 2 are used for this purpose to minimize confounding caused by potential repetition effects. The magnitude of the correlation coefficient is interpreted based on the lower bound of the 95% CI of r as follows (Andresen, 2000; Fitzpatrick, Davey, Buxton, & Jones, 1998; Kahn, Ohlendorf, Olsen, & Gordon, 2020): Excellent (0.6-1), Adequate (0.3-0.59) and Poor (<0.3).

2.3 Validity against Force Plate Data

Validity against the force plate outcomes is evaluated using Pearson's product-moment correlation coefficient (r). Initially, 22 outcomes are computed from the force plate data using the standard pipeline packaged with the force plate software. To minimize redundancy, a factor analysis is conducted to identify a minimum set of outcome domains (no. of factors) which explain 90% of the variance in the force plate outcomes. Afterwards, a single representative outcome is chosen from each of the domains. The selection of an outcome from within a domain is based on the simplicity of its interpretation. These representative outcomes are then used to evaluate the validity of the App outcomes. App outcomes from the static tests which are conducted concurrently to the force plate tests are used for this purpose to minimize time-related confounding.

App outcomes from ML and AP directions are only evaluated against force plate outcomes from the same directions. The magnitude of the correlation coefficient is interpreted based on the lower bound of the 95% CI of r as follows (Andresen, 2000; Fitzpatrick et al., 1998; Kahn et al., 2020): Excellent (0.6-1), Adequate (0.3-0.59) and Poor (<0.3).

2.4 Responsiveness to Repetition Effect and Differences across Tasks

Responsiveness of the outcomes to repetition effects across tests within a single task and to differences across tasks is evaluated using separate 1-way repeated measure ANOVAs with sphericity correction. For differences across tasks, data from the third test is used. Pair-wise comparisons are performed with Yuen's trimmed means test along with false discovery rate correction.

2.5 Assumptions

Normality and homogeneity of variance assumptions for data and the model residuals are evaluated with QQ-plots and fitted-vs-residuals plots where applicable. Statistical significance level is set at 0.05.

3 Results

3.1 Reliability of Gait Outcomes

Outcome	Qual	SEM	SEM%	ICC	Reliability
Gait.symmetry	HF	3.00	5	0.65 [0.5, 0.76]	M
Step.length	HF	0.02	3	0.94 [0.91, 0.96]	E
Step.time	HF	0.03	5	0.84 [0.76, 0.89]	H
Step.length.var	HF	4.00	77	0.16 [-0.06, 0.36]	P
Step.time.var	HF	3.00	47	0.51 [0.33, 0.65]	P
Step.length.asym	HF	2.00	56	0.6 [0.44, 0.72]	P
Step.time.asym	HF	2.00	47	0.68 [0.55, 0.78]	M
Step.velocity	HF	0.06	6	0.89 [0.84, 0.93]	H
Gait.symmetry	HT	4.00	7	0.63 [0.47, 0.74]	P
Step.length	HT	0.02	4	0.92 [0.88, 0.95]	H
Step.time	HT	0.04	5	0.84 [0.76, 0.89]	H
Step.length.var	HT	4.00	62	0.25 [0.03, 0.44]	P
Step.time.var	HT	4.00	54	0.4 [0.21, 0.57]	P
Step.length.asym	HT	3.00	62	0.59 [0.44, 0.72]	P
Step.time.asym	HT	3.00	66	0.52 [0.35, 0.66]	P
Step.velocity	HT	0.05	6	0.93 [0.89, 0.96]	H

3.2 Reliability of Outcomes for Static Tasks

Outcome	Task	Qual	SEM	SEM%	ICC	Reliability
Steadiness	Firm	EO	0.2	7	0.43 [0.23, 0.59]	P
Steadiness	Firm	EC	0.1	4	0.72 [0.6, 0.81]	M
Steadiness	Compliant	EO	0.2	5	0.62 [0.47, 0.74]	P
Steadiness	Compliant	EC	0.1	4	0.75 [0.51, 0.86]	M
Steadiness.ML	Firm	EO	0.3	7	0.38 [0.18, 0.55]	P
Steadiness.ML	Firm	EC	0.2	4	0.71 [0.59, 0.8]	M
Steadiness.ML	Compliant	EO	0.2	5	0.56 [0.37, 0.7]	P
Steadiness.ML	Compliant	EC	0.2	4	0.7 [0.45, 0.83]	P
Steadiness.AP	Firm	EO	0.2	5	0.52 [0.35, 0.66]	P
Steadiness.AP	Firm	EC	0.2	4	0.69 [0.56, 0.79]	M
Steadiness.AP	Compliant	EO	0.2	5	0.6 [0.45, 0.72]	P
Steadiness.AP	Compliant	EC	0.2	4	0.71 [0.49, 0.83]	P

3.3 Validity against Clinical Outcomes

3.3.1 Validity of Gait Outcomes

Task	Outcome	Clinical Outcome	r [95% CI]	Correlation
HF	Gait.symmetry	BBS	0.27 [0.05, 0.46]	Poor
HF	Gait.symmetry	FRT	-0.03 [-0.24, 0.19]	Poor
HF	Gait.symmetry	TUG	-0.43 [-0.59, -0.24]	Poor
HF	Step.length	BBS	0.45 [0.26, 0.6]	Poor
HF	Step.length	FRT	0.22 [0.01, 0.42]	Poor
HF	Step.length	TUG	-0.56 [-0.69, -0.39]	Adequate
HF	Step.time	BBS	-0.47 [-0.62, -0.28]	Poor
HF	Step.time	FRT	0.09 [-0.13, 0.3]	Poor
HF	Step.time	TUG	0.58 [0.42, 0.71]	Adequate
HF	Step.length.var	BBS	-0.2 [-0.4, 0.01]	Poor
HF	Step.length.var	FRT	0.13 [-0.09, 0.34]	Poor
HF	Step.length.var	TUG	0.21 [-0.01, 0.41]	Poor
HF	Step.time.var	BBS	-0.26 [-0.45, -0.05]	Poor
HF	Step.time.var	FRT	0.05 [-0.17, 0.26]	Poor
HF	Step.time.var	TUG	0.31 [0.1, 0.49]	Poor
HF	Step.length.asym	BBS	-0.17 [-0.37, 0.05]	Poor
HF	Step.length.asym	FRT	0.03 [-0.19, 0.24]	Poor
HF	Step.length.asym	TUG	0.04 [-0.17, 0.26]	Poor
HF	Step.time.asym	BBS	-0.15 [-0.35, 0.07]	Poor
HF	Step.time.asym	FRT	0.23 [0.02, 0.43]	Poor
HF	Step.time.asym	TUG	0.01 [-0.2, 0.23]	Poor
HF	Step.velocity	BBS	0.55 [0.38, 0.69]	Adequate
HF	Step.velocity	FRT	0.04 [-0.17, 0.26]	Poor
HF	Step.velocity	TUG	-0.7 [-0.79, -0.57]	Adequate
HT	Gait.symmetry	BBS	0.41 [0.21, 0.57]	Poor
HT	Gait.symmetry	FRT	-0.06 [-0.28, 0.15]	Poor
HT	Gait.symmetry	TUG	-0.47 [-0.62, -0.28]	Poor
HT	Step.length	BBS	0.53 [0.36, 0.67]	Adequate
HT	Step.length	FRT	0.16 [-0.06, 0.36]	Poor
HT	Step.length	TUG	-0.58 [-0.7, -0.41]	Adequate
HT	Step.time	BBS	-0.26 [-0.45, -0.05]	Poor
HT	Step.time	FRT	0.15 [-0.07, 0.35]	Poor
HT	Step.time	TUG	0.5 [0.32, 0.65]	Adequate
HT	Step.length.var	BBS	-0.38 [-0.55, -0.18]	Poor
HT	Step.length.var	FRT	0.22 [0, 0.41]	Poor
HT	Step.length.var	TUG	0.33 [0.12, 0.51]	Poor
HT	Step.time.var	BBS	-0.47 [-0.63, -0.29]	Poor
HT	Step.time.var	FRT	0.14 [-0.08, 0.34]	Poor
HT	Step.time.var	TUG	0.46 [0.28, 0.62]	Poor
HT	Step.length.asym	BBS	-0.37 [-0.54, -0.17]	Poor
HT	Step.length.asym	FRT	-0.06 [-0.27, 0.16]	Poor
HT	Step.length.asym	TUG	0.16 [-0.06, 0.36]	Poor
HT	Step.time.asym	BBS	-0.31 [-0.49, -0.1]	Poor
HT	Step.time.asym	FRT	0.17 [-0.04, 0.37]	Poor
HT	Step.time.asym	TUG	0.23 [0.01, 0.42]	Poor
HT	Step.velocity	BBS	0.51 [0.33, 0.66]	Adequate
HT	Step.velocity	FRT	0.01 [-0.2, 0.23]	Poor
HT	Step.velocity	TUG	-0.67 [-0.77, -0.53]	Adequate

3.3.2 Validity of Outcomes for Static Tasks

Task	Qual	Outcome	Clinical Outcome	r [95% CI]	Correlation
Firm	EO	Steadiness	BBS	-0.15 [-0.36, 0.06]	Poor
Firm	EO	Steadiness	FRT	-0.13 [-0.34, 0.09]	Poor
Firm	EO	Steadiness	TUG	0.19 [-0.02, 0.39]	Poor
Firm	EC	Steadiness	BBS	-0.15 [-0.36, 0.06]	Poor
Firm	EC	Steadiness	FRT	-0.19 [-0.39, 0.03]	Poor
Firm	EC	Steadiness	TUG	-0.02 [-0.24, 0.19]	Poor
Compliant	EO	Steadiness	BBS	-0.13 [-0.33, 0.09]	Poor
Compliant	EO	Steadiness	FRT	-0.13 [-0.34, 0.09]	Poor
Compliant	EO	Steadiness	TUG	0.02 [-0.19, 0.24]	Poor
Compliant	EC	Steadiness	BBS	0.08 [-0.14, 0.29]	Poor
Compliant	EC	Steadiness	FRT	0.07 [-0.15, 0.28]	Poor
Compliant	EC	Steadiness	TUG	-0.19 [-0.39, 0.03]	Poor
Firm	EO	Steadiness.ML	BBS	-0.11 [-0.32, 0.11]	Poor
Firm	EO	Steadiness.ML	FRT	-0.11 [-0.32, 0.11]	Poor
Firm	EO	Steadiness.ML	TUG	0.15 [-0.07, 0.36]	Poor
Firm	EC	Steadiness.ML	BBS	-0.08 [-0.29, 0.14]	Poor
Firm	EC	Steadiness.ML	FRT	-0.19 [-0.39, 0.02]	Poor
Firm	EC	Steadiness.ML	TUG	-0.1 [-0.31, 0.12]	Poor
Compliant	EO	Steadiness.ML	BBS	-0.05 [-0.26, 0.17]	Poor
Compliant	EO	Steadiness.ML	FRT	-0.07 [-0.28, 0.15]	Poor
Compliant	EO	Steadiness.ML	TUG	-0.1 [-0.31, 0.12]	Poor
Compliant	EC	Steadiness.ML	BBS	0.16 [-0.06, 0.36]	Poor
Compliant	EC	Steadiness.ML	FRT	0.08 [-0.14, 0.29]	Poor
Compliant	EC	Steadiness.ML	TUG	-0.3 [-0.48, -0.09]	Poor
Firm	EO	Steadiness.AP	BBS	-0.14 [-0.34, 0.08]	Poor
Firm	EO	Steadiness.AP	FRT	-0.11 [-0.32, 0.11]	Poor
Firm	EO	Steadiness.AP	TUG	0.19 [-0.02, 0.39]	Poor
Firm	EC	Steadiness.AP	BBS	-0.13 [-0.33, 0.09]	Poor
Firm	EC	Steadiness.AP	FRT	-0.09 [-0.3, 0.13]	Poor
Firm	EC	Steadiness.AP	TUG	-0.02 [-0.23, 0.2]	Poor
Compliant	EO	Steadiness.AP	BBS	-0.1 [-0.31, 0.12]	Poor
Compliant	EO	Steadiness.AP	FRT	-0.13 [-0.34, 0.09]	Poor
Compliant	EO	Steadiness.AP	TUG	0.05 [-0.17, 0.26]	Poor
Compliant	EC	Steadiness.AP	BBS	0.09 [-0.13, 0.3]	Poor
Compliant	EC	Steadiness.AP	FRT	0.09 [-0.13, 0.3]	Poor
Compliant	EC	Steadiness.AP	TUG	-0.15 [-0.35, 0.07]	Poor

3.4 Validity againt Force Plate Data

3.4.1 Selection of Force Plate Outcomes

A five factor model is chosen as it explains 90% of the data variance. A single outcome is chosen to represent each factor for further analysis. These outcomes are: TotalMLSway, TotalAPSway, TotalDistanceWandered, MinStepDistance and MaxStepDistance.

```
In smc, smcs < 0 were set to .0
In smc, smcs < 0 were set to .0
In smc, smcs < 0 were set to .0

Loading required namespace: GPArotation

In factor.scores, the correlation matrix is singular, the pseudo inverse is  used

                           MR1       MR2       MR3       MR5        MR4
SS loadings          6.8677327 4.3344501 3.8971556 2.6191441 2.15417272
Proportion Var       0.3121697 0.1970205 0.1771434 0.1190520 0.09791694
Cumulative Var       0.3121697 0.5091901 0.6863336 0.8053856 0.90330251
Proportion Explained  0.3455871 0.2181113 0.1961064 0.1317964 0.10839884
Cumulative Proportion 0.3455871 0.5636983 0.7598048 0.8916012 1.00000000
```

Factor Model for the Force Plate Outcomes

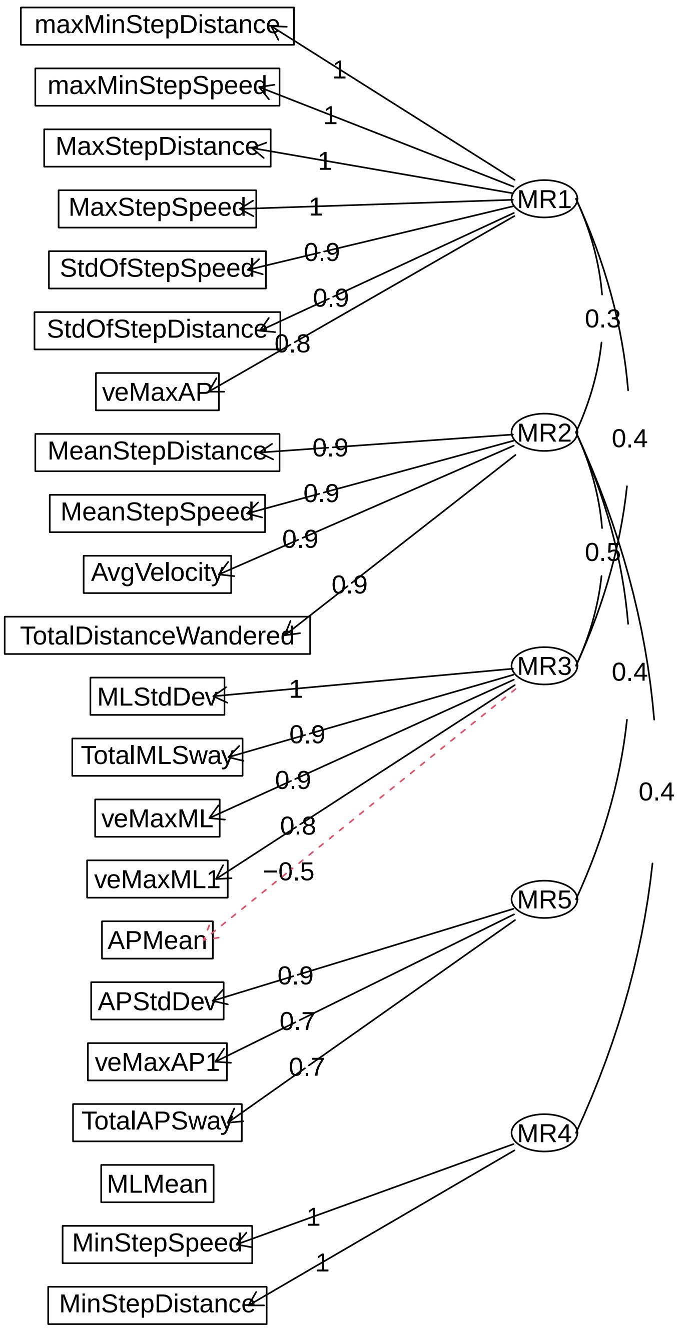

3.4.2 Validity of Outcomes for Static Tasks

Task	Qual	Outcome	FP Outcome	r [95% CI]	Correlation
Firm	EO	Steadiness	TotalDistanceWandered	-0.7 [-0.8, -0.55]	Adequate
Firm	EO	Steadiness	MinStepDistance	-0.54 [-0.68, -0.35]	Adequate
Firm	EO	Steadiness	MaxStepDistance	-0.53 [-0.68, -0.34]	Adequate
Firm	EC	Steadiness	TotalDistanceWandered	-0.83 [-0.89, -0.73]	Excellent
Firm	EC	Steadiness	MinStepDistance	-0.57 [-0.71, -0.39]	Adequate
Firm	EC	Steadiness	MaxStepDistance	-0.7 [-0.8, -0.55]	Adequate
Compliant	EO	Steadiness	TotalDistanceWandered	-0.73 [-0.82, -0.59]	Adequate
Compliant	EO	Steadiness	MinStepDistance	-0.31 [-0.51, -0.08]	Poor
Compliant	EO	Steadiness	MaxStepDistance	-0.02 [-0.26, 0.22]	Poor
Compliant	EC	Steadiness	TotalDistanceWandered	-0.9 [-0.94, -0.85]	Excellent
Compliant	EC	Steadiness	MinStepDistance	-0.29 [-0.49, -0.05]	Poor
Compliant	EC	Steadiness	MaxStepDistance	-0.83 [-0.89, -0.74]	Excellent
Firm	EO	Steadiness.ML	TotalMLSway	-0.47 [-0.64, -0.27]	Poor
Firm	EC	Steadiness.ML	TotalMLSway	-0.6 [-0.73, -0.42]	Adequate
Compliant	EO	Steadiness.ML	TotalMLSway	-0.31 [-0.51, -0.08]	Poor
Compliant	EC	Steadiness.ML	TotalMLSway	-0.59 [-0.72, -0.41]	Adequate
Firm	EO	Steadiness.AP	TotalAPSway	-0.34 [-0.53, -0.11]	Poor
Firm	EC	Steadiness.AP	TotalAPSway	-0.46 [-0.63, -0.25]	Poor
Compliant	EO	Steadiness.AP	TotalAPSway	-0.15 [-0.37, 0.09]	Poor
Compliant	EC	Steadiness.AP	TotalAPSway	-0.58 [-0.72, -0.4]	Adequate

3.5 Responsiveness to Repetition Effect and Task Differences

3.5.1 Responsiveness of Gait Outcomes

Outcome	Qual	Mean±SD	rmANOVA
Gait.symmetry	HF	64±5, 64±5	F(1, 82)=0.11, 0.739
Gait.symmetry	HT	59±7, 60±7	F(1, 82)=0.97, 0.327
Gait.symmetry	Between		F(1, 82)=48.29, <0.001
Step.length	HF	0.57±0.07, 0.57±0.07	F(1, 82)=0.23, 0.631
Step.length	HT	0.54±0.08, 0.55±0.09	F(1, 82)=1.86, 0.176
Step.length	Between		F(1, 82)=36.88, <0.001
Step.time	HF	0.64±0.08, 0.63±0.08	F(1, 82)=0.57, 0.453
Step.time	HT	0.67±0.09, 0.66±0.09	F(1, 82)=2.41, 0.125
Step.time	Between		F(1, 82)=18.37, <0.001
Step.length.var	HF	6±4, 6±6	F(1, 82)=0, 0.997
Step.length.var	HT	7±4, 7±6	F(1, 82)=0.01, 0.93
Step.length.var	Between		F(1, 82)=11.56, 0.001
Step.time.var	HF	7±4, 6±5	F(1, 82)=0.05, 0.829
Step.time.var	HT	8±5, 7±5	F(1, 82)=0.12, 0.729
Step.time.var	Between		F(1, 82)=3.44, 0.067
Step.length.asym	HF	4±4, 4±4	F(1, 82)=1.09, 0.299
Step.length.asym	HT	5±4, 5±4	F(1, 82)=1.29, 0.26
Step.length.asym	Between		F(1, 82)=0.08, 0.782
Step.time.asym	HF	4±4, 5±4	F(1, 82)=1.79, 0.184
Step.time.asym	HT	5±5, 5±5	F(1, 82)=0.89, 0.349
Step.time.asym	Between		F(1, 82)=1.89, 0.173
Step.velocity	HF	0.91±0.18, 0.91±0.18	F(1, 82)=0.71, 0.402
Step.velocity	HT	0.82±0.19, 0.85±0.2	F(1, 82)=10.4, 0.002
Step.velocity	Between		F(1, 82)=51.78, <0.001

3.5.2 Responsiveness of Outcomes for Static Tasks

Outcome	Task	Qual	Mean±SD	rmANOVA
Steadiness	Firm:EO	EO	3.56±0.32, 3.54±0.31	F(1, 82)=0.35, 0.555
Steadiness	Firm:EC	EC	3.54±0.27, 3.52±0.26	F(1, 82)=0.89, 0.347
Steadiness	Compliant:EO	EO	3.33±0.29, 3.39±0.29	F(1, 82)=5.17, 0.026
Steadiness	Compliant:EC	EC	3.13±0.32, 3.25±0.32	F(1, 82)=27.43, <0.001
Steadiness	Between:			F(2.45, 201.29)=46.67, <0.001
Steadiness.ML	Firm:EO	EO	4.22±0.37, 4.21±0.35	F(1, 82)=0.07, 0.797
Steadiness.ML	Firm:EC	EC	4.26±0.31, 4.25±0.29	F(1, 82)=0.09, 0.761
Steadiness.ML	Compliant:EO	EO	3.93±0.31, 4.04±0.32	F(1, 82)=12.79, 0.001
Steadiness.ML	Compliant:EC	EC	3.8±0.35, 3.94±0.35	F(1, 82)=27.96, <0.001
Steadiness.ML	Between:			F(2.38, 195.31)=36.74, <0.001
Steadiness.AP	Firm:EO	EO	4.17±0.28, 4.14±0.29	F(1, 82)=1.02, 0.316
Steadiness.AP	Firm:EC	EC	4.08±0.28, 4.07±0.26	F(1, 82)=0.58, 0.448
Steadiness.AP	Compliant:EO	EO	3.97±0.3, 4.01±0.29	F(1, 82)=2.12, 0.149
Steadiness.AP	Compliant:EC	EC	3.69±0.32, 3.81±0.31	F(1, 82)=22.63, <0.001
Steadiness.AP	Between:			F(2.61, 214.1)=54.25, <0.001

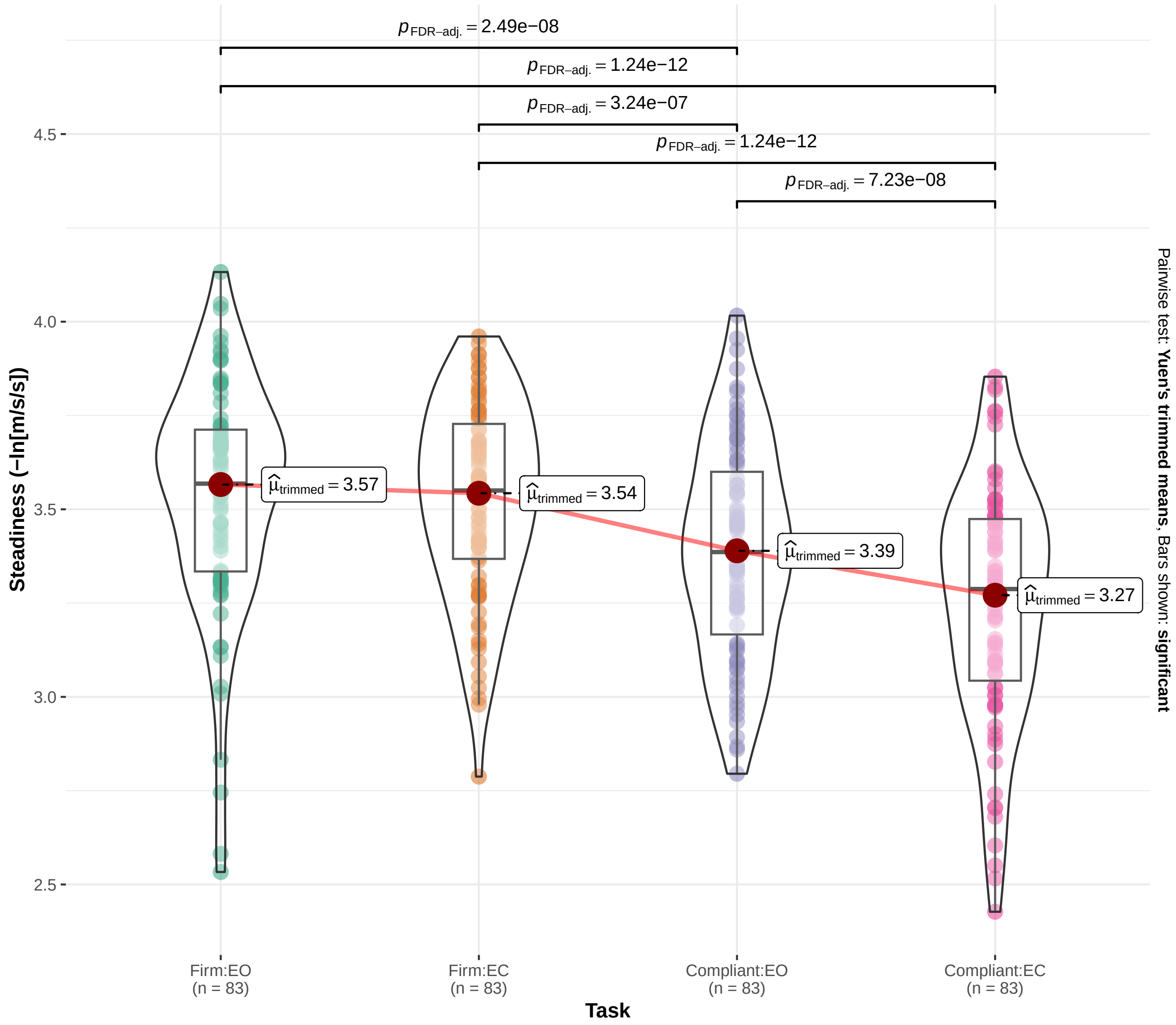

$p_{\text{FDR-adj.}} = 2.49\text{e}{-08}$
$p_{\text{FDR-adj.}} = 1.24\text{e}{-12}$
$p_{\text{FDR-adj.}} = 3.24\text{e}{-07}$
$p_{\text{FDR-adj.}} = 1.24\text{e}{-12}$
$p_{\text{FDR-adj.}} = 7.23\text{e}{-08}$
$\hat{\mu}_{\text{trimmed}} = 3.57$
$\hat{\mu}_{\text{trimmed}} = 3.54$
$\hat{\mu}_{\text{trimmed}} = 3.39$
$\hat{\mu}_{\text{trimmed}} = 3.27$
Steadiness (−ln[m/s/s])
4.5
4.0
3.5
3.0
2.5
Firm:EO (n = 83)
Firm:EC (n = 83)
Compliant:EO (n = 83)
Compliant:EC (n = 83)
Task
Pairwise test: **Yuen's trimmed means**, Bars shown: **significant**

4 Additional Results

4.1 Model Residuals: Reliability of Gait Outcomes

4.1.1 Gait.symmetry, HF

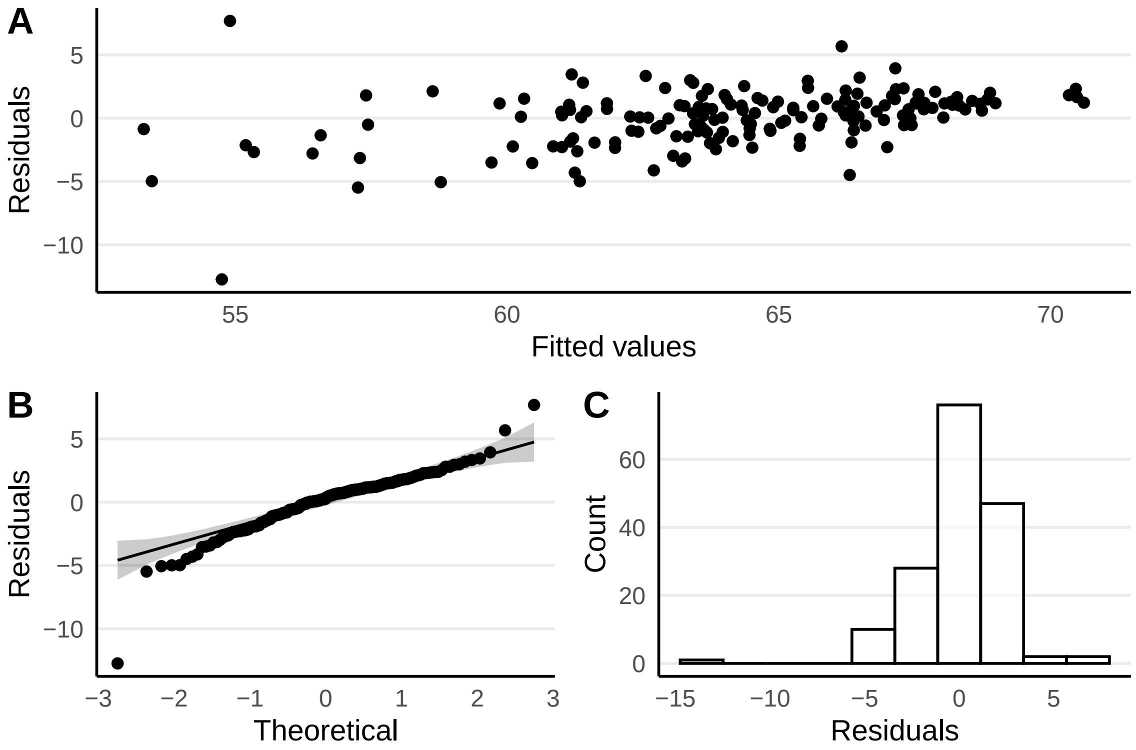

4.1.2 Step.length, HF

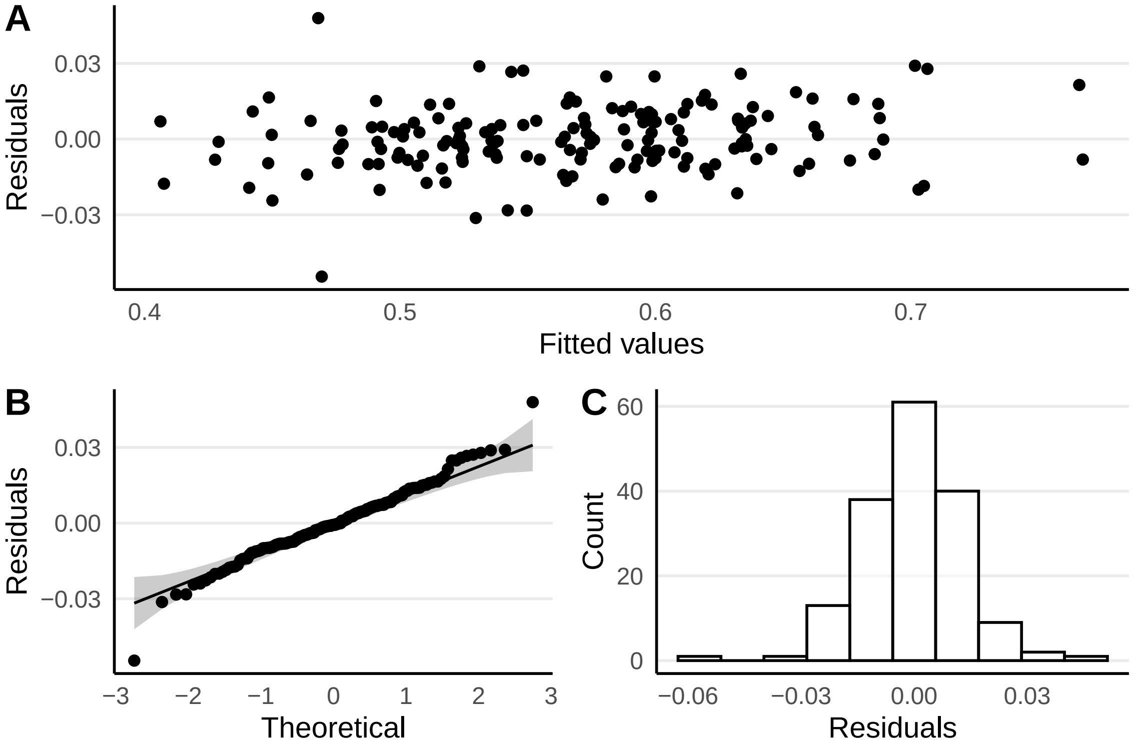

4.1.3 Step.time, HF

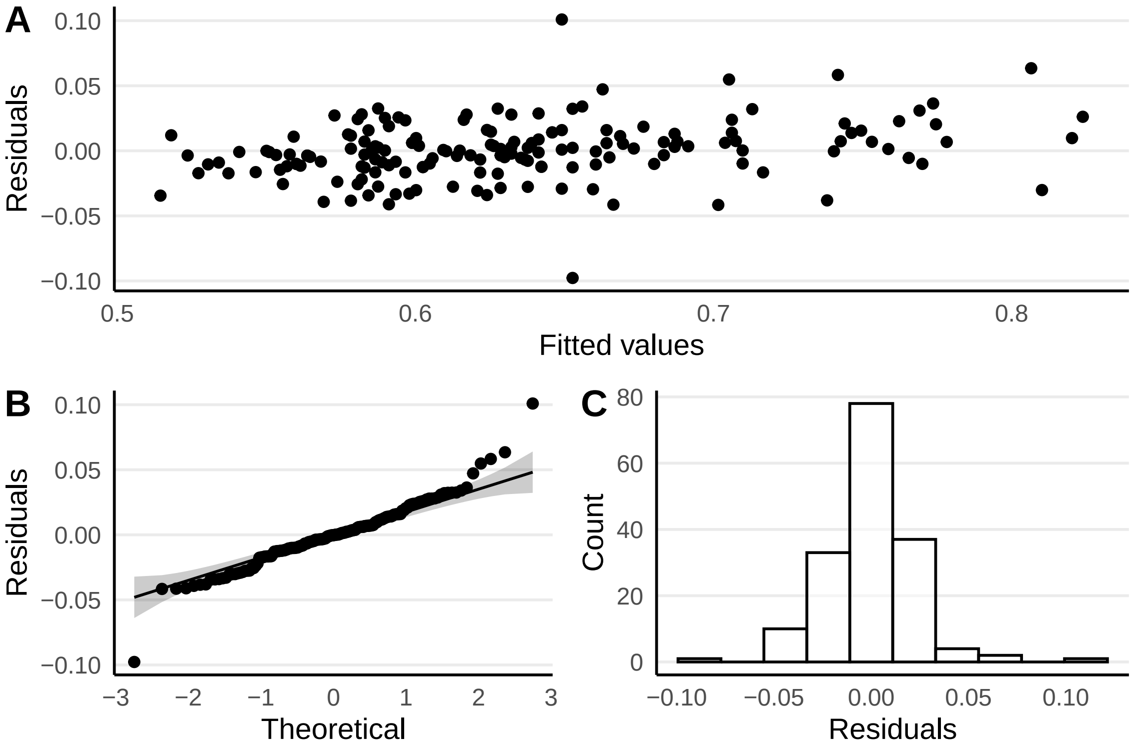

4.1.4 Step.length.var, HF

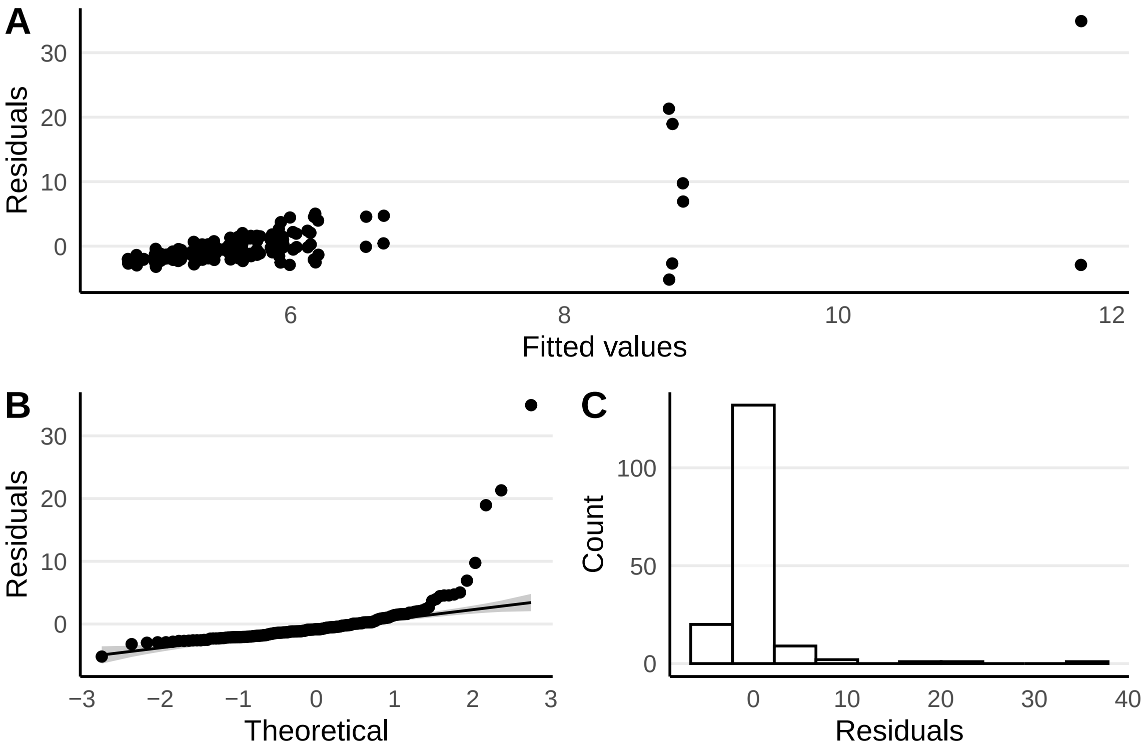

4.1.5 Step.time.var, HF

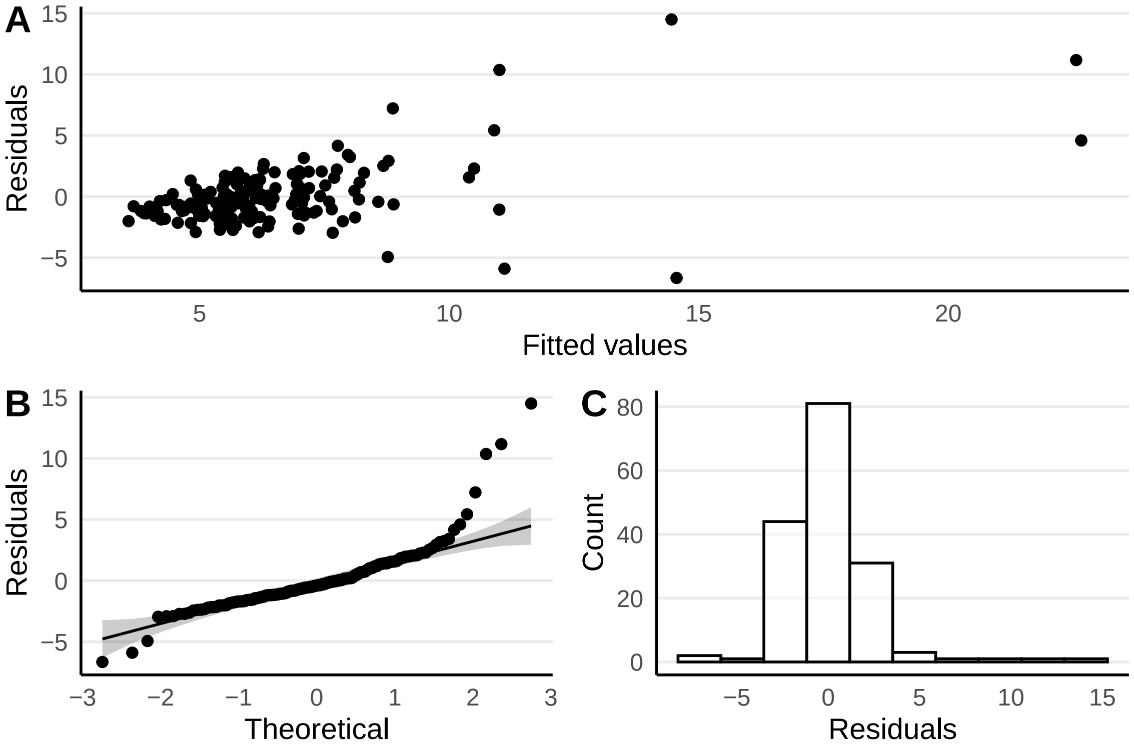

4.1.6 Step.length.asym, HF

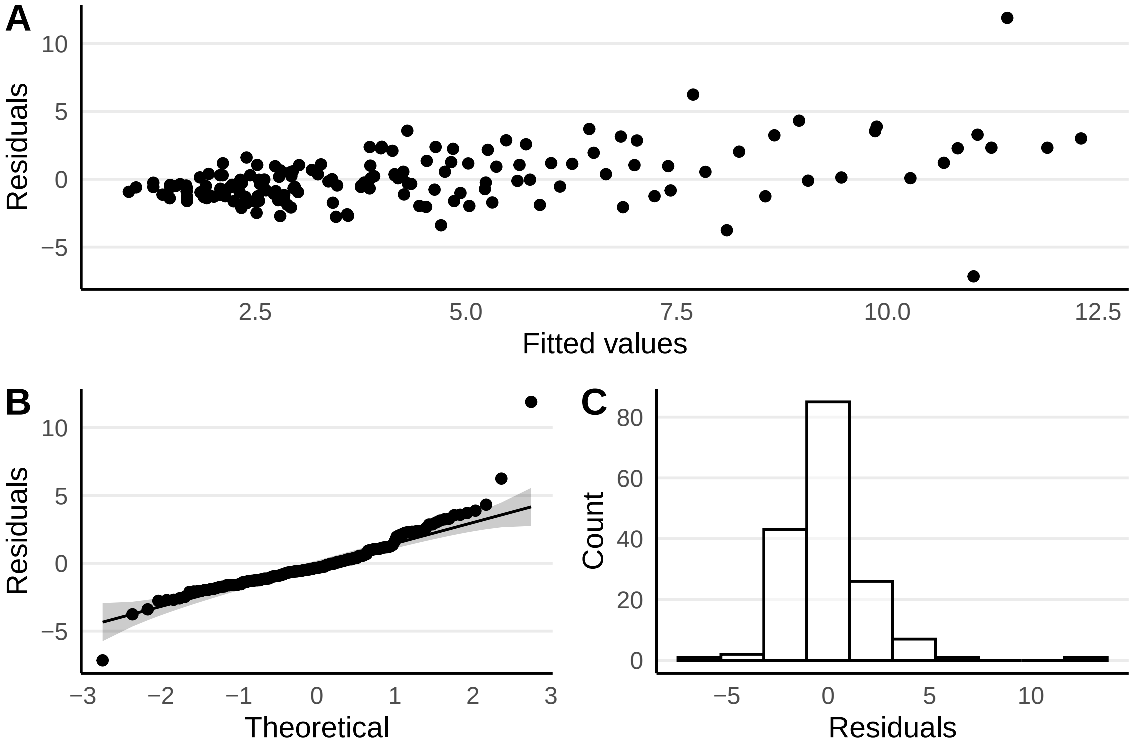

4.1.7 Step.time.asym, HF

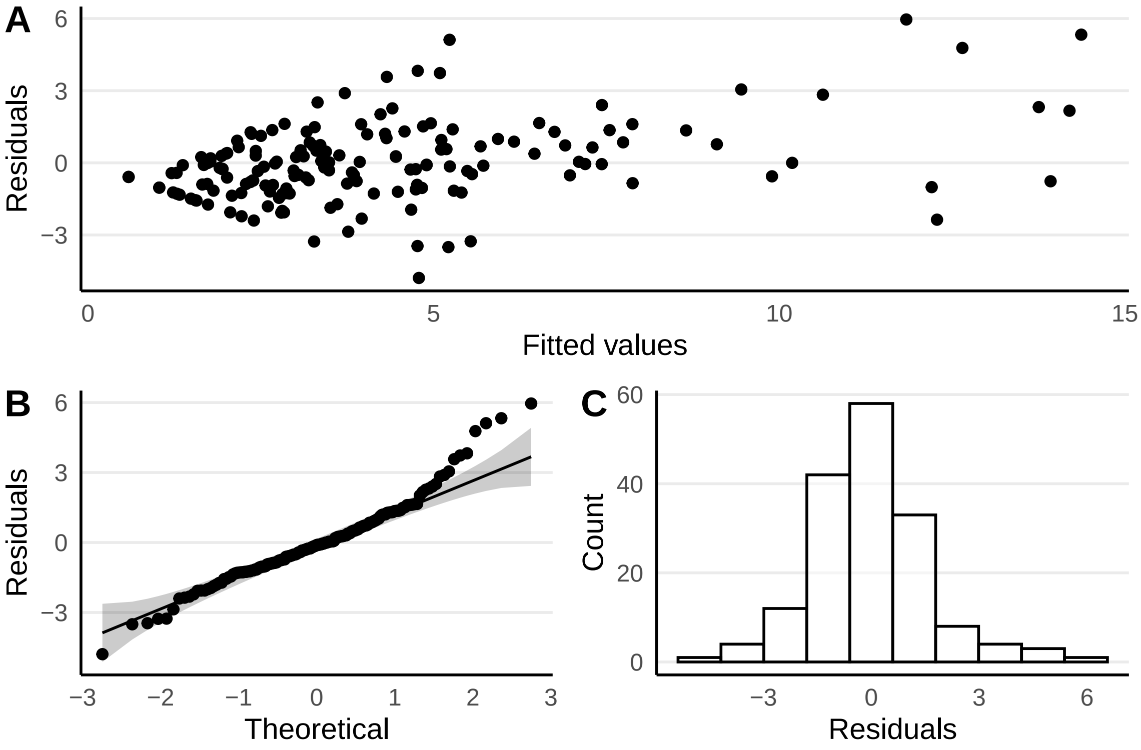

4.1.8 Step.velocity, HF

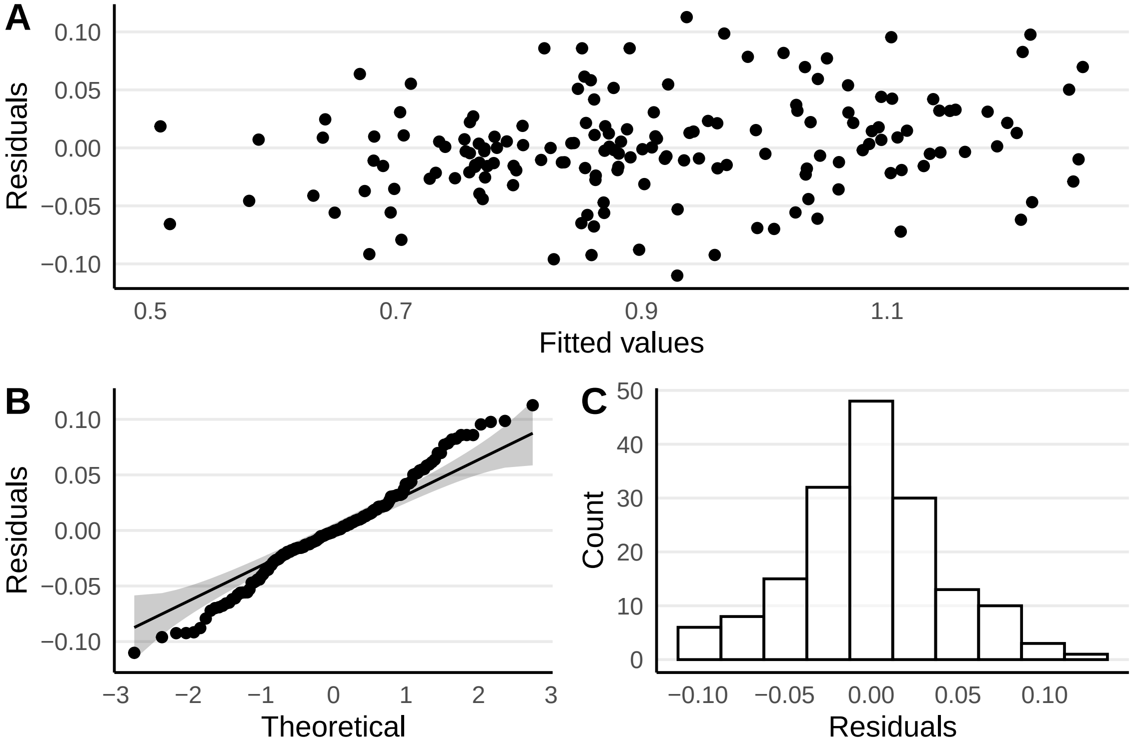

4.1.9 Gait.symmetry, HT

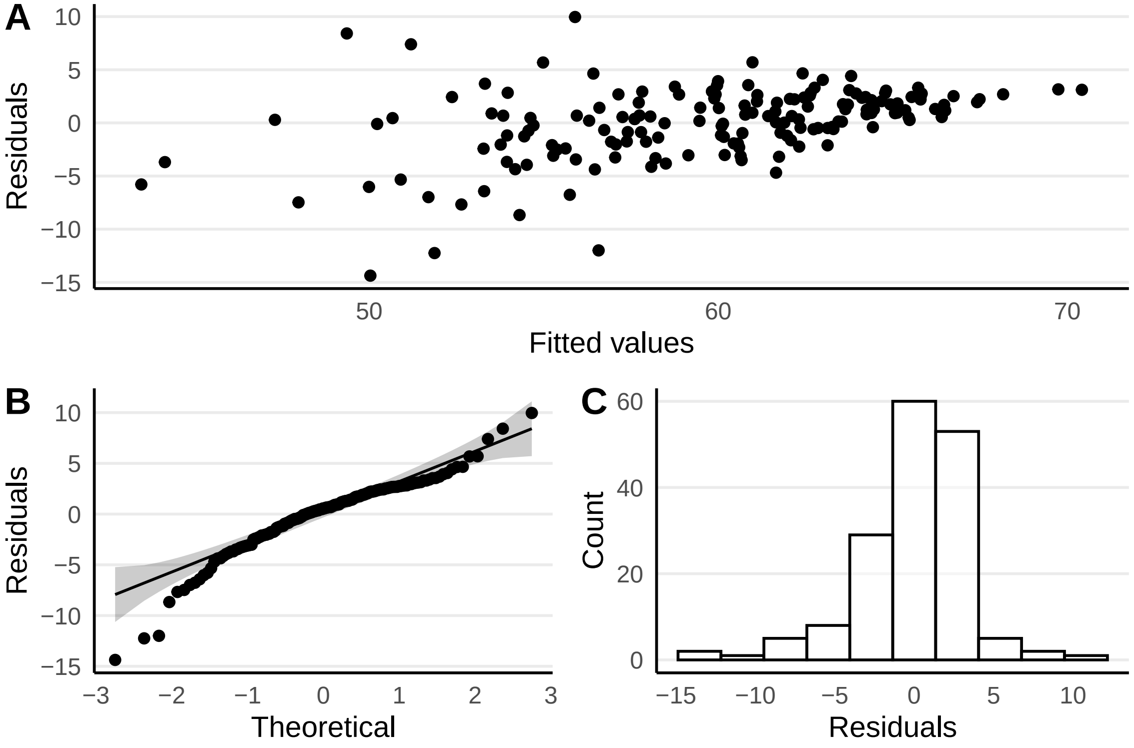

4.1.10 Step.length, HT

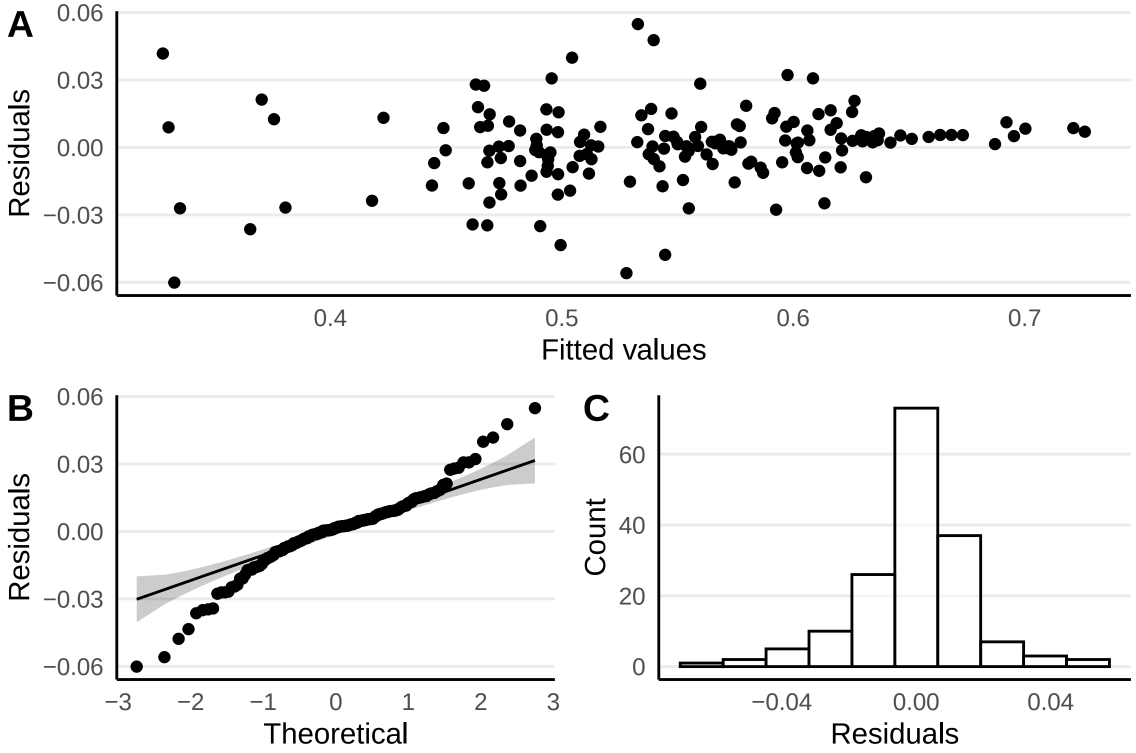

4.1.11 Step.time, HT

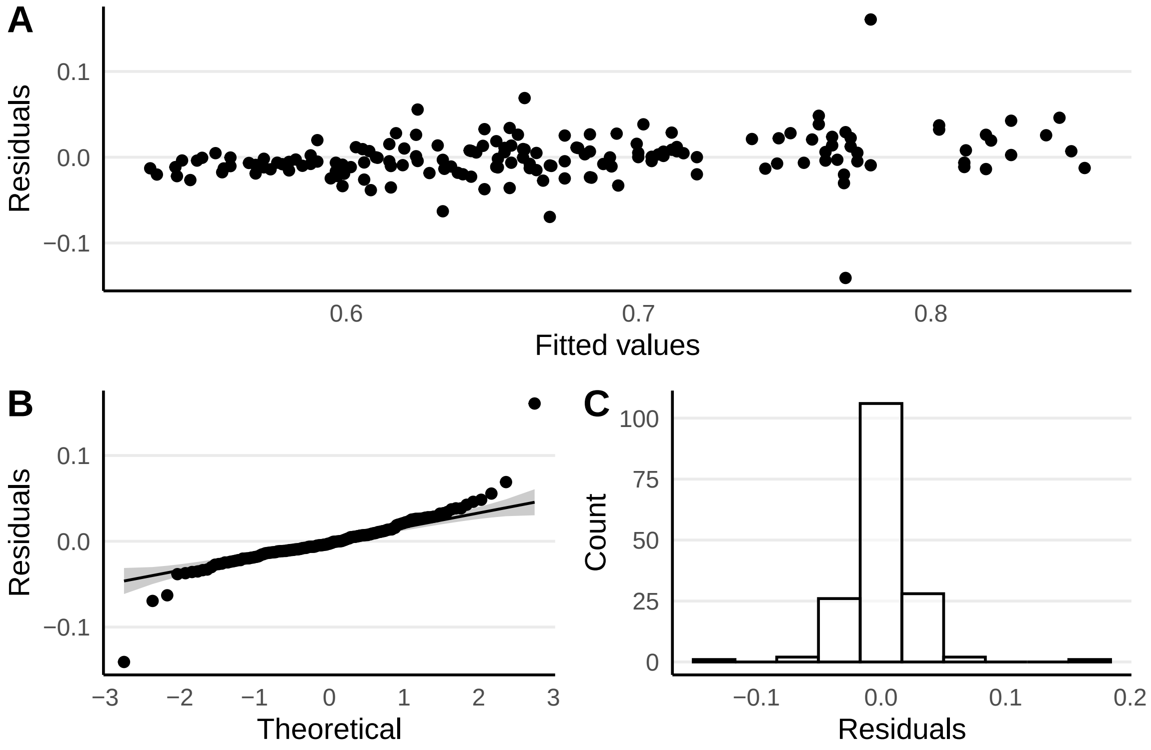

4.1.12 Step.length.var, HT

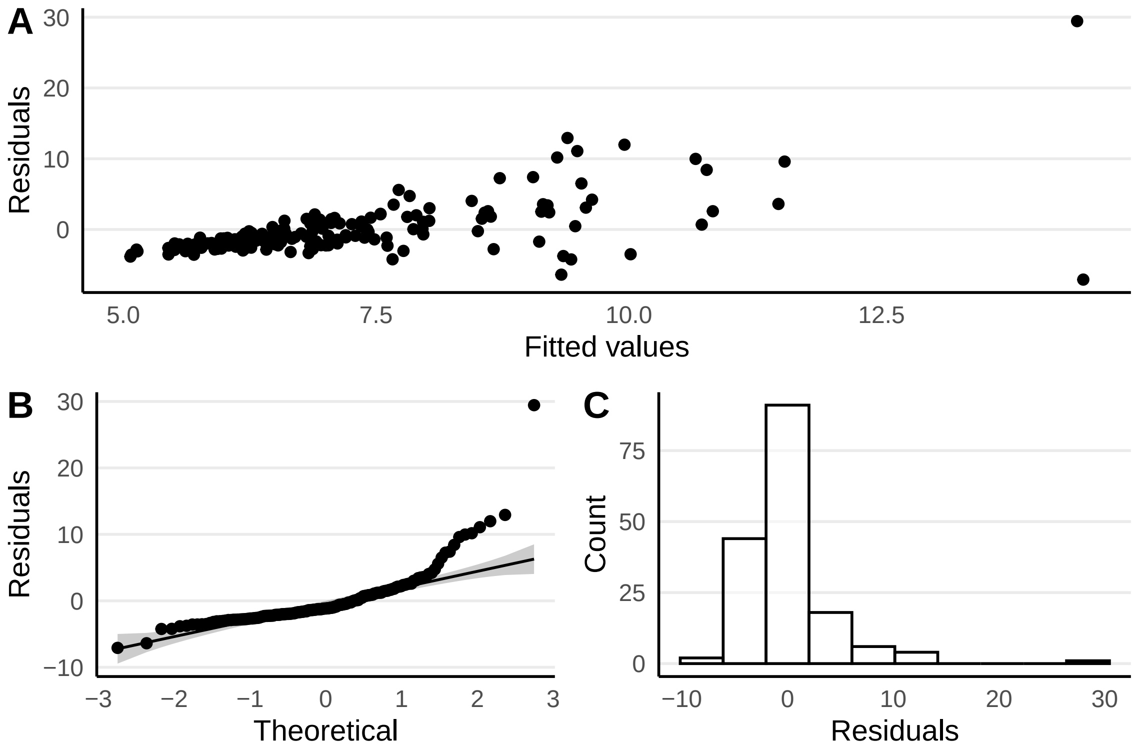

4.1.13 Step.time.var, HT

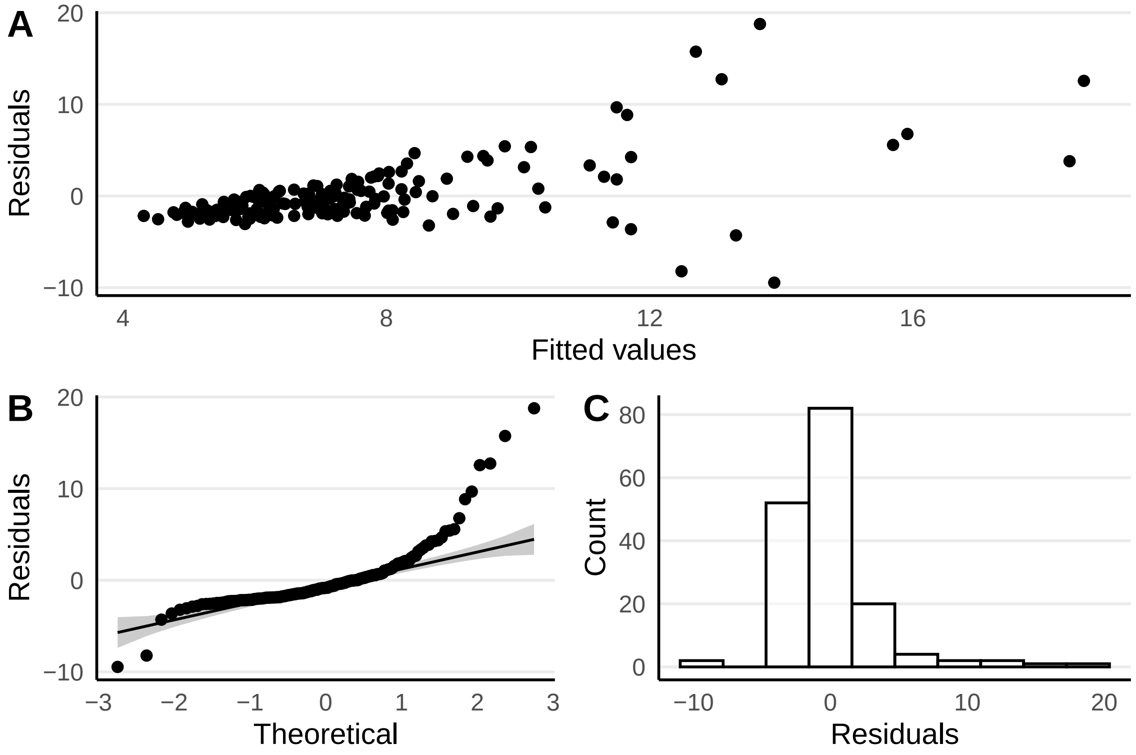

4.1.14 Step.length.asym, HT

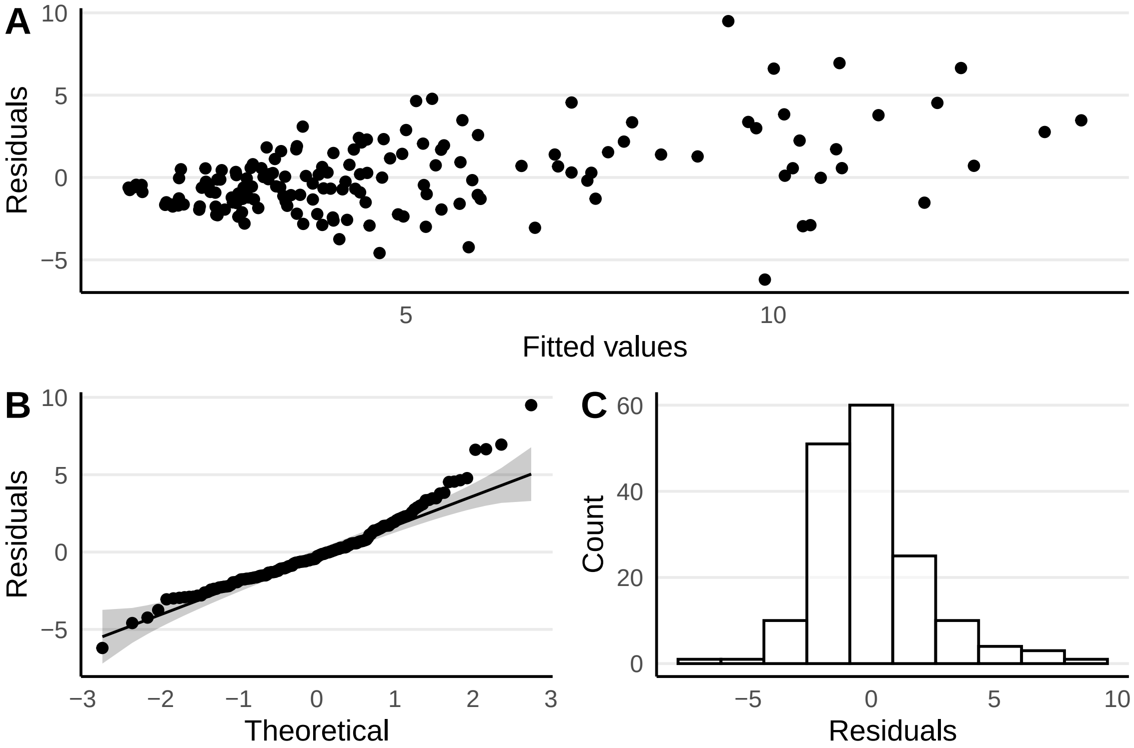

4.1.15 Step.time.asym, HT

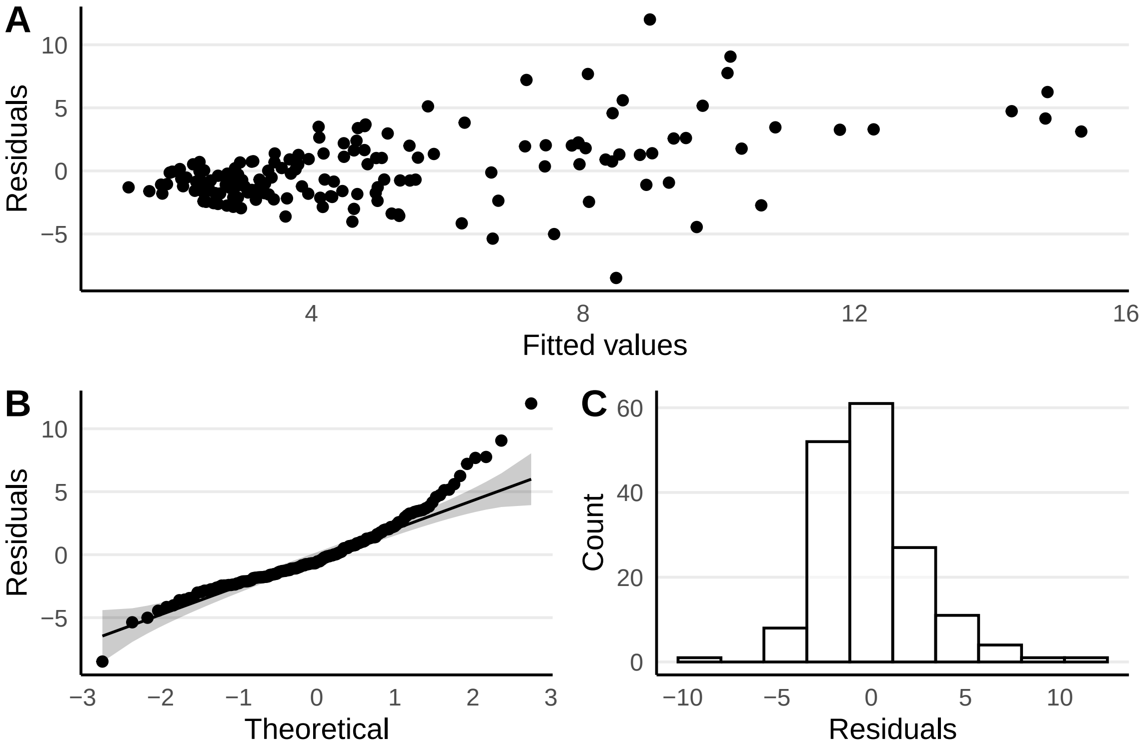

4.1.16 Step.velocity, HT

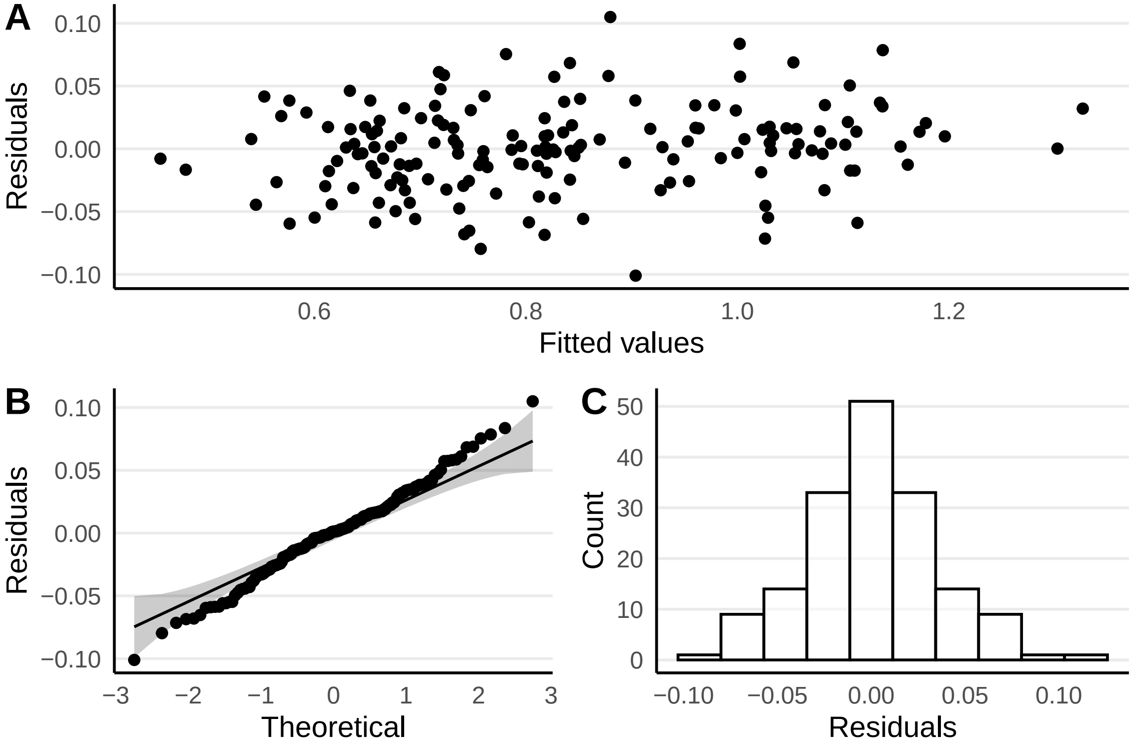

4.2 Model Residuals: Reliability of Outcomes for Static Tasks

4.2.1 Steadiness, Firm EO

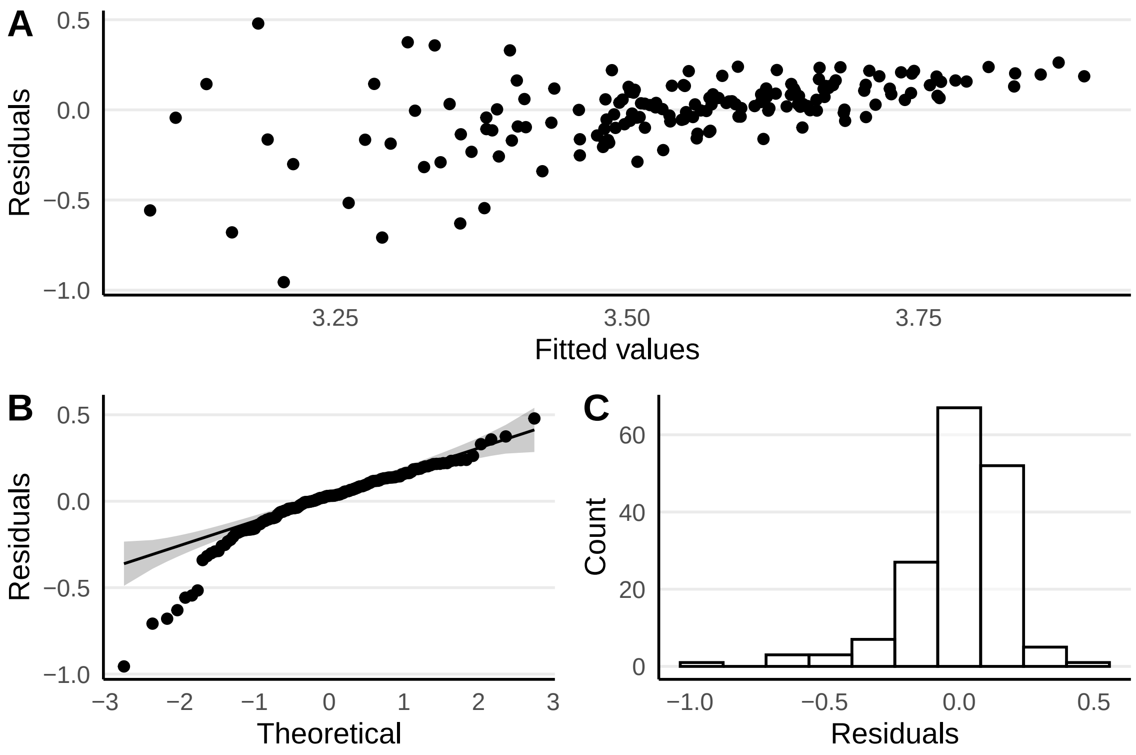

4.2.2 Steadiness, Firm EC

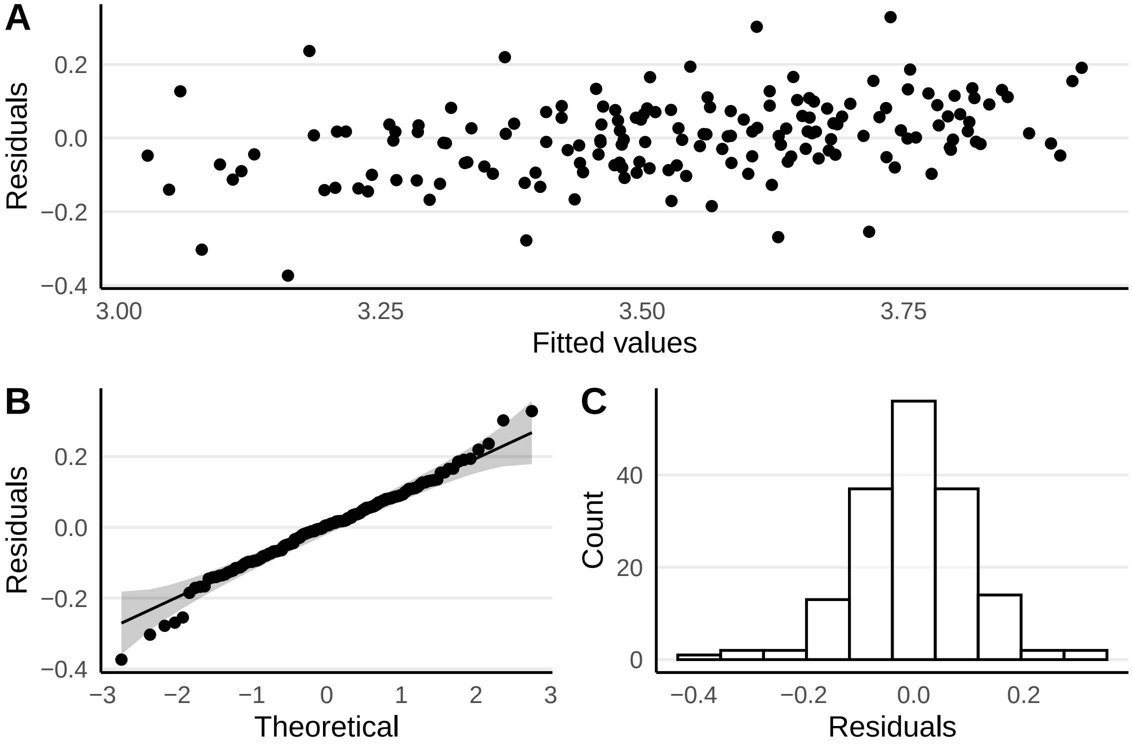

4.2.3 Steadiness, Compliant EO

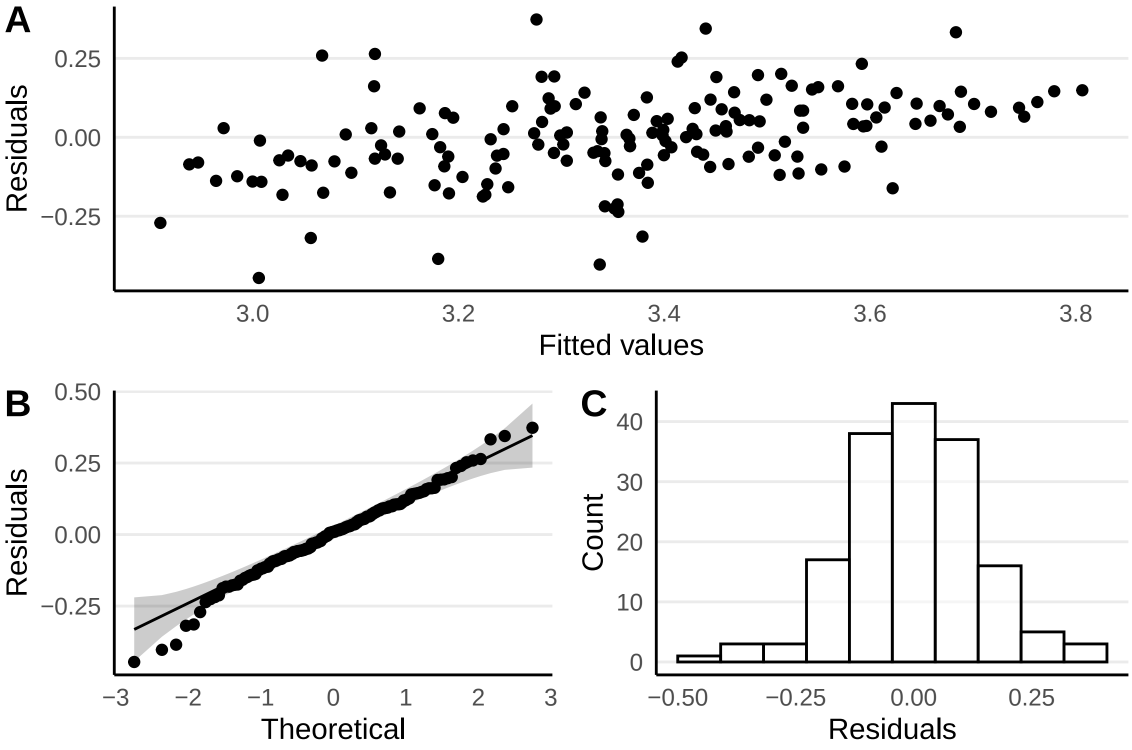

4.2.4 Steadiness, Compliant EC

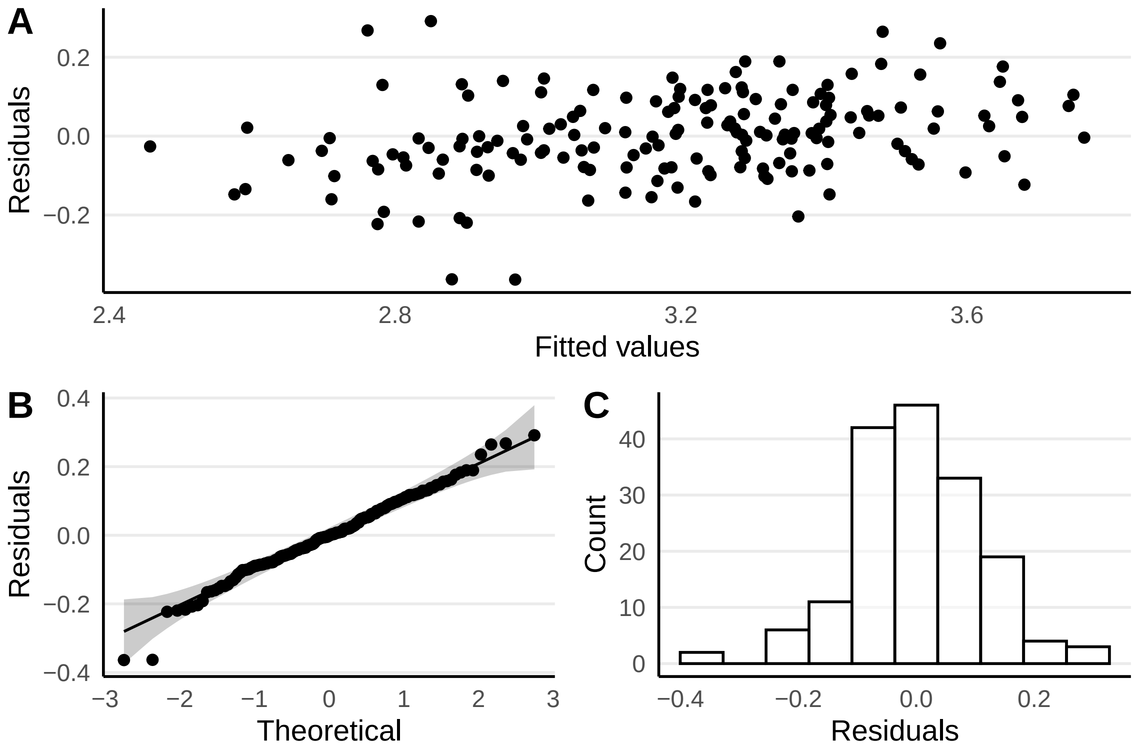

4.2.5 Steadiness.ML, Firm EO

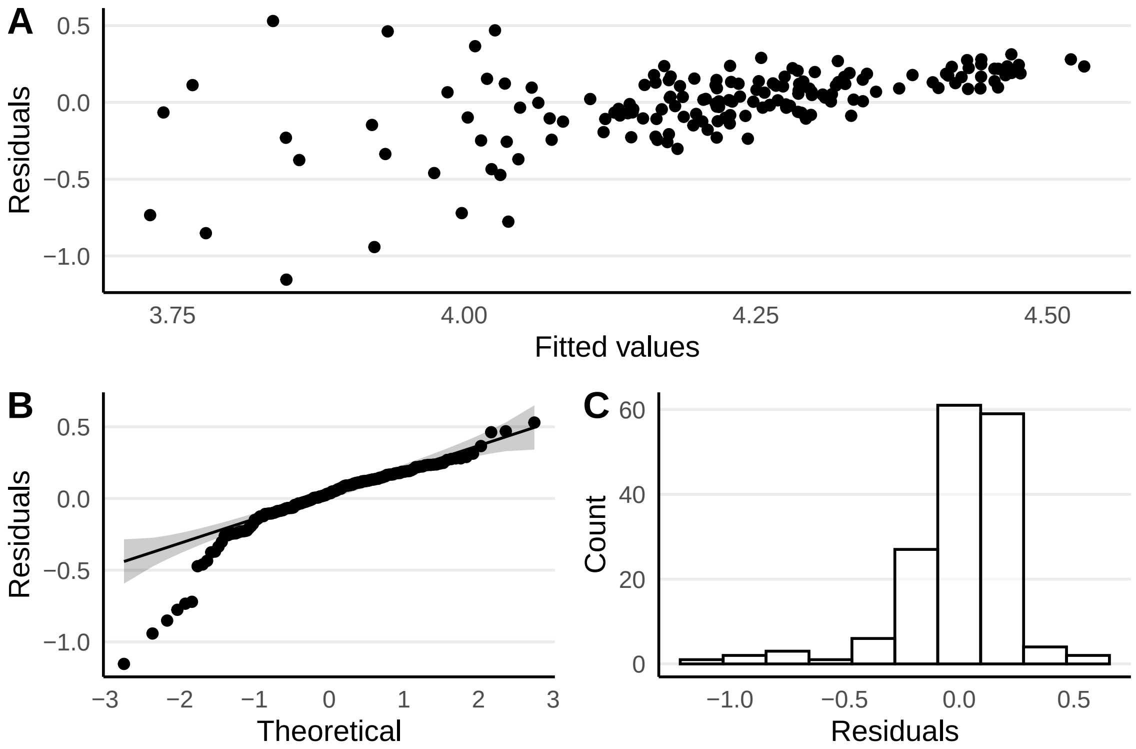

4.2.6 Steadiness.ML, Firm EC

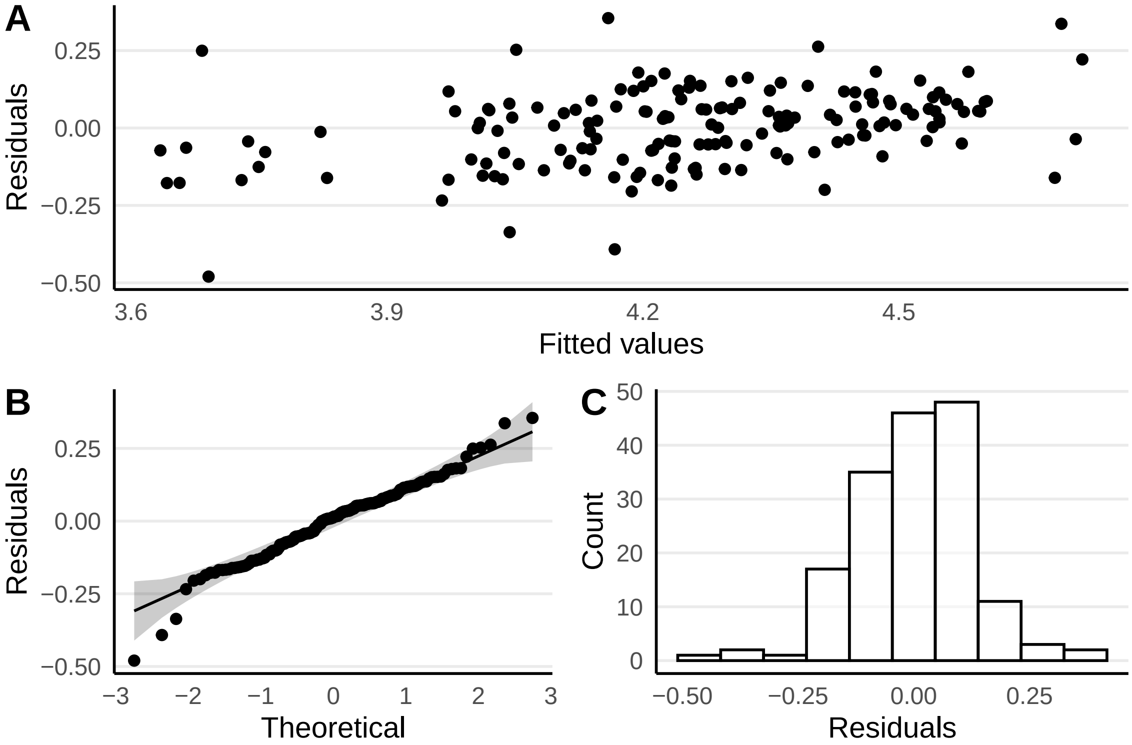

4.2.7 Steadiness.ML, Compliant EO

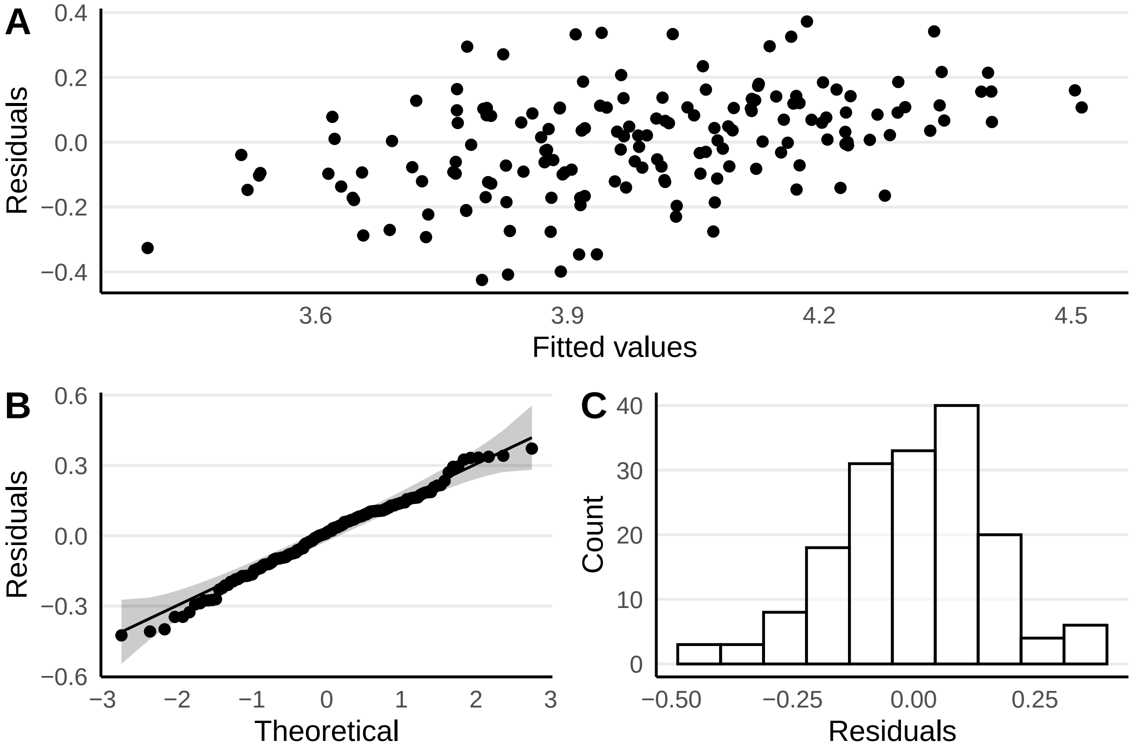

4.2.8 Steadiness.ML, Compliant EC

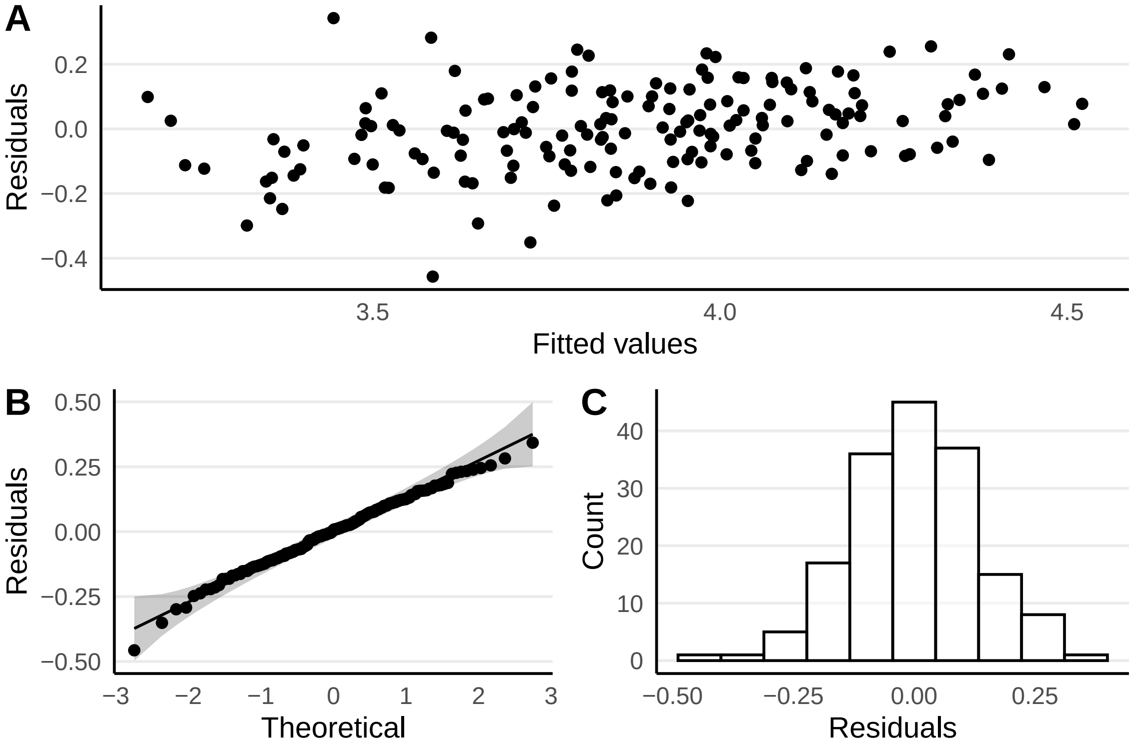

4.2.9 Steadiness.AP, Firm EO

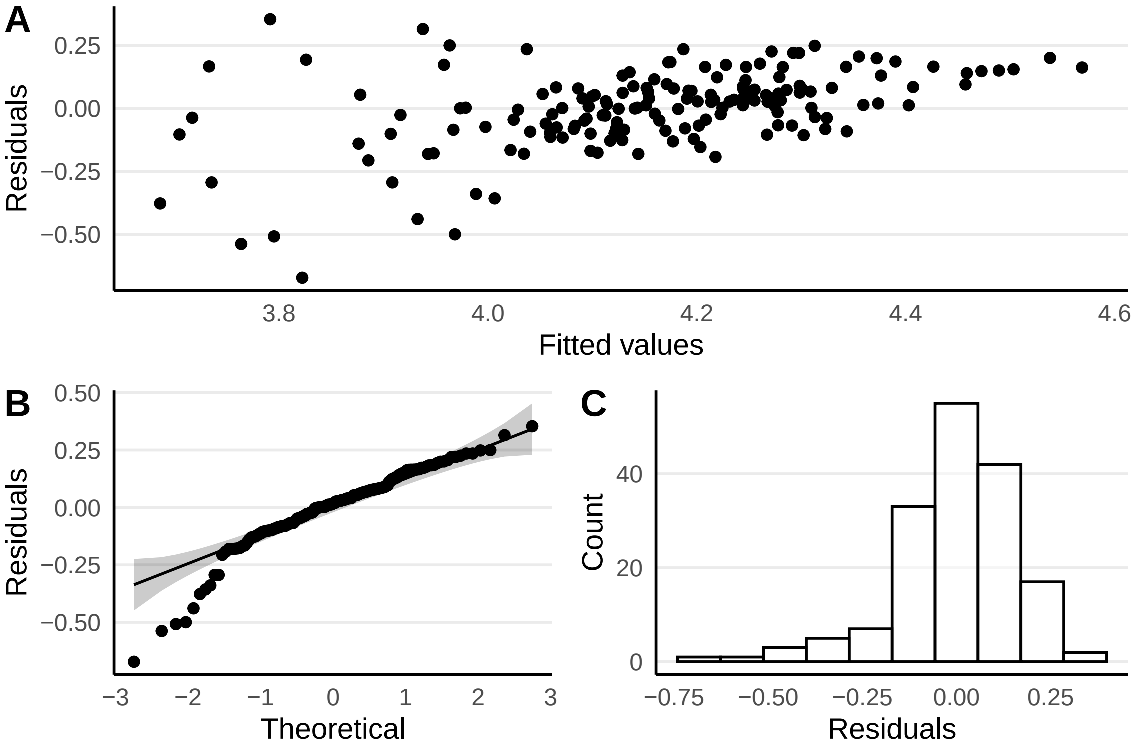

4.2.10 Steadiness.AP, Firm EC

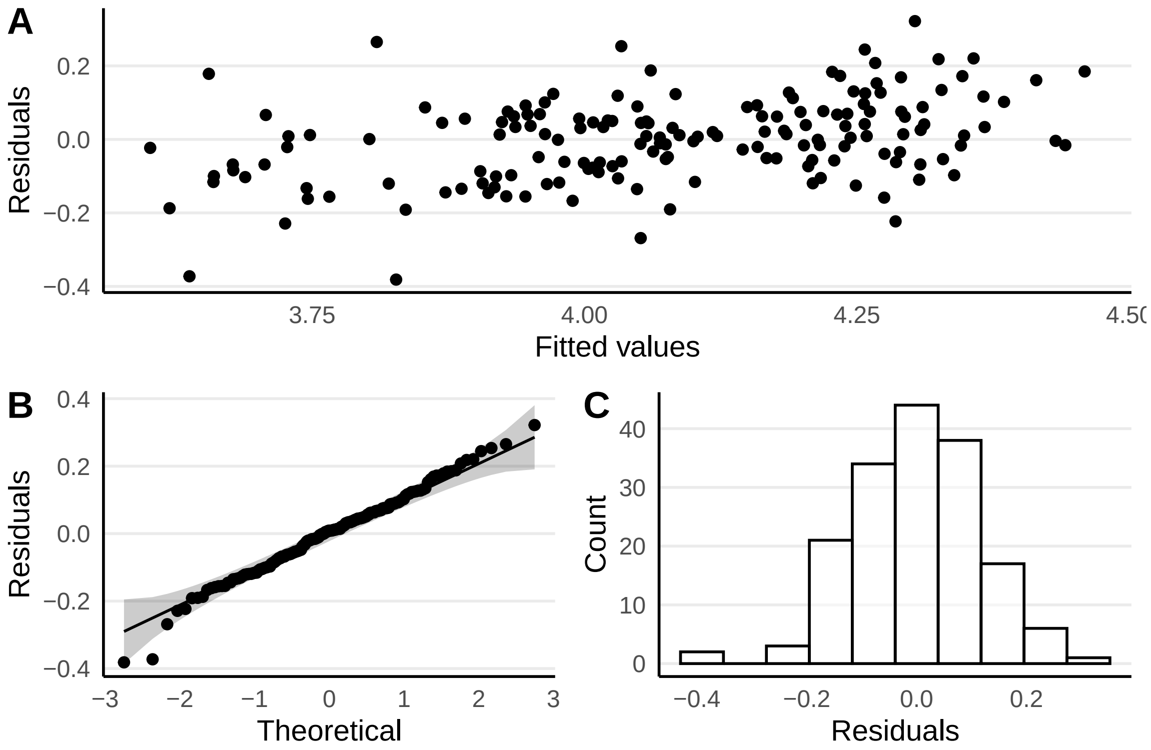

4.2.11 Steadiness.AP, Compliant EO

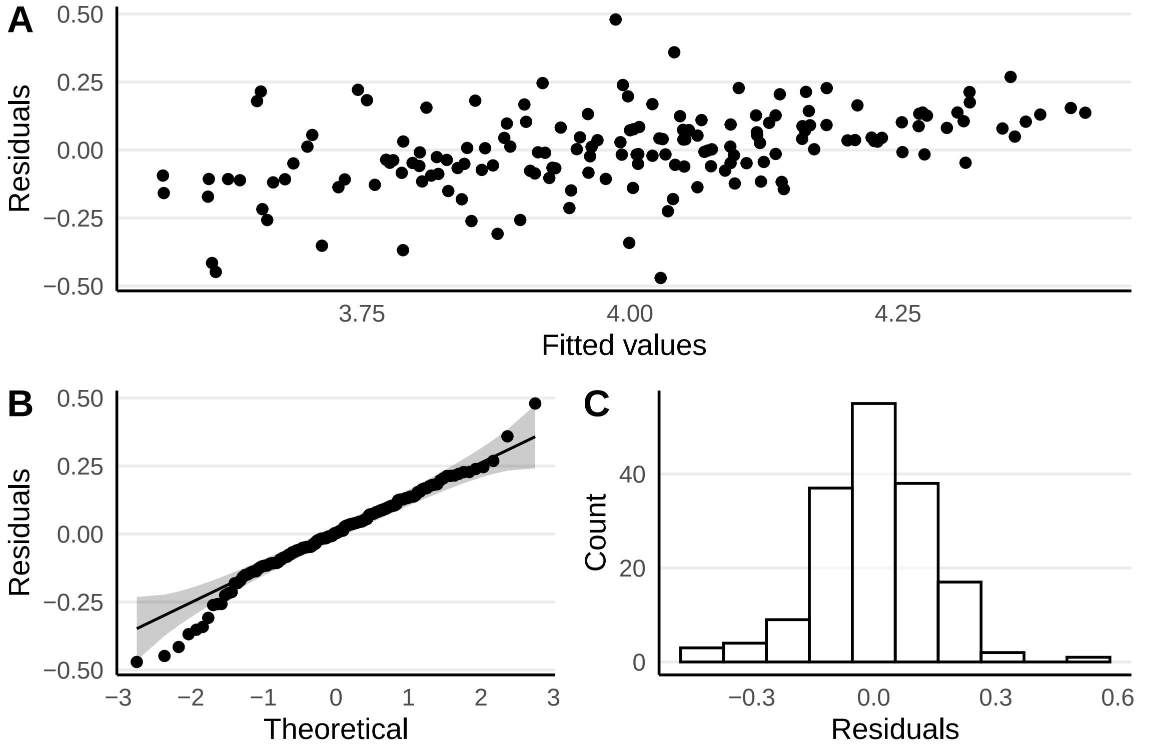

4.2.12 Steadiness.AP, Compliant EC

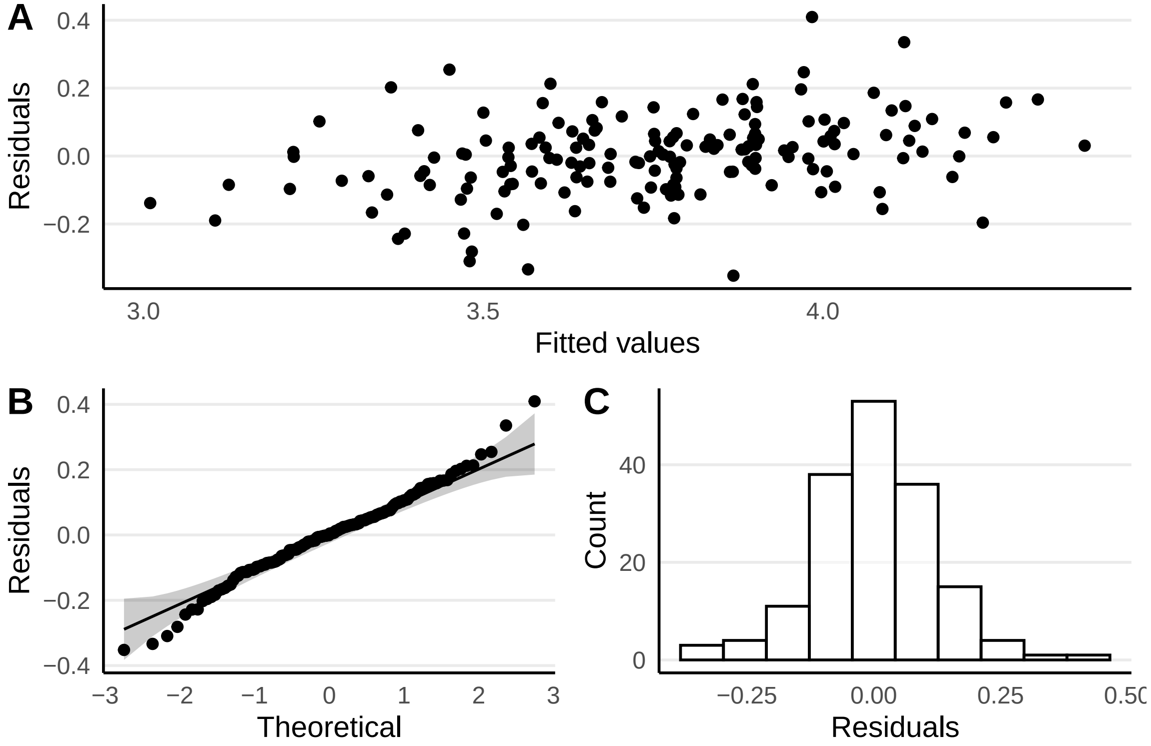

References

Andresen, E. M. (2000). Criteria for assessing the tools of disability outcomes research. *Archives of Physical Medicine and Rehabilitation*, *81*, S15–S20. https://doi.org/10.1053/apmr.2000.20619

Fitzpatrick, R., Davey, C., Buxton, M. J., & Jones, D. R. (1998). *Evaluating patient-based outcome measures for use in clinical trials. 2.* https://doi.org/10.3310/hta2140

Kahn, J. H., Ohlendorf, A., Olsen, A., & Gordon, K. E. (2020). Reliability and validity of the functional gait assessment in incomplete spinal cord injury. *Topics in Spinal Cord Injury Rehabilitation*, *26*(4), 268–274. https://doi.org/10.46292/sci19-00069

Koo, T. K., & Li, M. Y. (2016). A guideline of selecting and reporting intraclass correlation coefficients for reliability research. *Journal of Chiropractic Medicine*, *15*(2), 155–163. https://doi.org/10.1016/j.jcm.2016.02.012

Munro, B. (2005). *Statistical methods for health care research* (Vol. 1). Philadelphia: Lippincott Williams & Wilkins.

Nakagawa, S., Johnson, P. C., & Schielzeth, H. (2017). The coefficient of determination r 2 and intra-class correlation coefficient from generalized linear mixed-effects models revisited and expanded. *Journal of the Royal Society Interface*, *14*(134), 20170213. https://doi.org/10.1098/rsif.2017.0213

Nakagawa, S., & Schielzeth, H. (2010). Repeatability for gaussian and non-gaussian data: A practical guide for biologists. *Biological Reviews*, *85*(4), 935–956. https://doi.org/10.1111/j.1469-185x.2010.00141.x

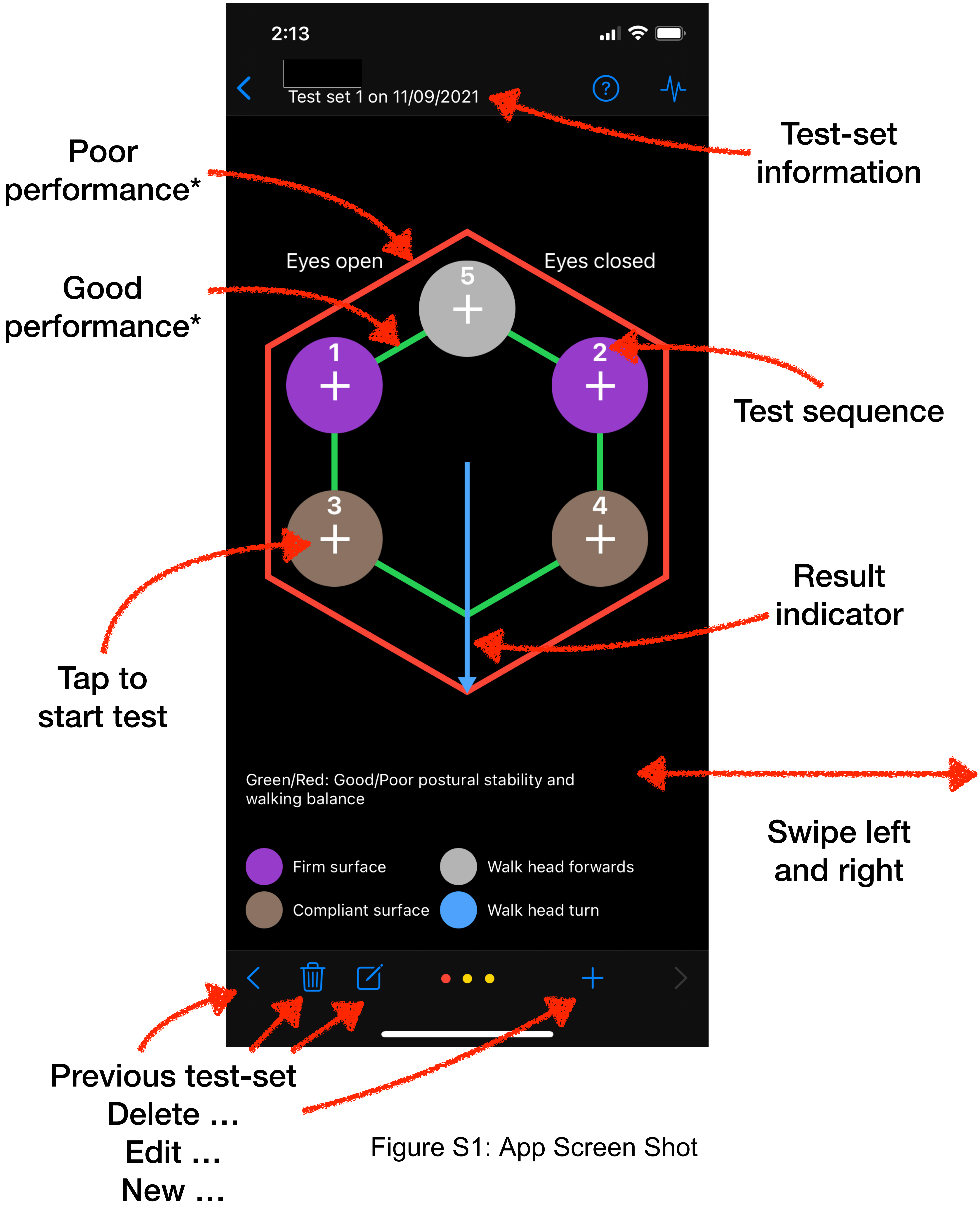

Figure S1: App Screen Shot
